# Overexpression of Lin28A in neural progenitor cells in vivo does not lead to brain tumor formation but results in reduced spine density

**DOI:** 10.1186/s40478-021-01289-1

**Published:** 2021-11-20

**Authors:** Maximilian Middelkamp, Lisa Ruck, Christoph Krisp, Piotr Sumisławski, Behnam Mohammadi, Matthias Dottermusch, Valerie Meister, Lukas Küster, Hartmut Schlüter, Sabine Windhorst, Julia E. Neumann

**Affiliations:** 1 Center for Molecular Neurobiology Hamburg (ZMNH), University Medical Center Hamburg-Eppendorf, Falkenried 94, 20251 Hamburg, Germany; 2 Institute of Neuropathology, University Medical Center Hamburg-Eppendorf, 20251 Hamburg, Germany; 3 Institute of Clinical Chemistry and Laboratory Medicine, Mass Spectrometric Proteomics, University Medical Center Hamburg-Eppendorf, 20251 Hamburg, Germany; 4 Institute of Neuropathology, Ludwig-Maximilians-University Munich, 81377 Munich, Germany; 5 Department of Biochemistry and Signal Transduction, University Medical Center Hamburg-Eppendorf, 20251 Hamburg, Germany

**Keywords:** LIN28A, Embryonal tumor, ETMR, Proteomics, Spine density, Microtubule, mTOR

## Abstract

LIN28A overexpression has been identified in malignant brain tumors called embryonal tumors with multilayered rosettes (ETMR) but its specific role during brain development remains largely unknown. Radial glia cells of the ventricular zone (VZ) are proposed as a cell of origin for ETMR. We asked whether an overexpression of LIN28A in such cells might affect brain development or result in the formation of brain tumors.

Constitutive overexpression of LIN28A in *hGFAP-cre::lsl-Lin28A* (GL) mice led to a transient increase of proliferation in the cortical VZ at embryonic stages but no postnatal brain tumor formation. Postnatally, GL mice displayed a pyramidal cell layer dispersion of the hippocampus and altered spine and dendrite morphology, including reduced dendritic spine densities in the hippocampus and cortex. GL mice displayed hyperkinetic activity and differential quantitative MS-based proteomics revealed altered time dependent molecular functions regarding mRNA processing and spine morphogenesis. Phosphoproteomic analyses indicated a downregulation of mTOR pathway modulated proteins such as Map1b being involved in microtubule dynamics.

In conclusion, we show that Lin28A overexpression transiently increases proliferation of neural precursor cells but it is not sufficient to drive brain tumors in vivo*.* In contrast, Lin28A impacts on protein abundancy patterns related to spine morphogenesis and phosphorylation levels of proteins involved in microtubule dynamics, resulting in decreased spine densities of neurons in the hippocampus and cortex as well as in altered behavior. Our work provides new insights into the role of LIN28A for neuronal morphogenesis and development and may reveal future targets for treatment of ETMR patients*.*

**Supplementary Information:**

The online version contains supplementary material available at 10.1186/s40478-021-01289-1.

## Introduction

Lin28A is considered a stem cell marker, which is commonly known for being expressed in pluripotent progenitor cells [12, 76]. Under physiological conditions, expression of Lin28A is found during early embryonic and fetal development in diverse organs [55, 72]. Moreover, it is one of the factors necessary to induce pluripotent stem cells in vitro [76, 78]. As a RNA binding protein, Lin28A mostly occurs in the cytoplasm and consists of a cold shock domain and a zinc finger binding motif, affecting its RNA binding capacity [5]. Posttranscriptionally, Lin28A inhibits the *let-7*-microRNA family in embryonic stem cells [19, 44, 64]. Conversely,* let-7* is also able to suppress Lin28A via direct binding [11, 21].

The Lin28A-*let7*-axis has been described as an important regulator of glucose metabolism by interacting with the insulin-PI3K-mTOR pathway [81]. Relating to this, Lin28A was additionally identified mediating aerobic glycolysis and having an impact on cancer promotion [37, 40]. In line with this, LIN28A expression has been reported in different tumor entities, often being associated with increased malignancy [3, 69]. In brain tumors, LIN28A expression was described in gliomas [37], atypical teratoid/rhabdoid tumors (AT/RT) [50, 67] and embryonal tumors with multilayered rosettes (ETMR) [33, 56]. Particularly in ETMR, LIN28A overexpression is recognized as an immunohistochemical hallmark, in routine diagnostics [34, 50, 57]. During early brain development, Lin28A-mediated promotion of protein synthesis was described to be important for neuronal precursor cell proliferation and maintenance [22, 75] and in vitro studies indicated an implication in cell fate determination during glioneurogenesis [4, 75]. However, the specific role of LIN28A during embryonal and postnatal brain development and for brain tumor formation still remains unknown.

We asked whether an overexpression of Lin28A in neuronal progenitor cells in vivo might affect brain development or result in the formation of brain tumors. Previously, we have established a mouse model showing formation of embryonal tumors with multilayered rosettes (ETMR) like tumors by overexpressing Sonic hedgehog- and Wnt signaling in *hGFAP*-positive ventricular precursor cells [46]. Therefore, we chose to construct and analyze the characteristics of a *hGFAP-cre::lsl-Lin28A* (GL) mouse model.

## Results

### Overexpression of Lin28A in neural precursor cells does not lead to brain tumor formation.

**Fig. 1:**
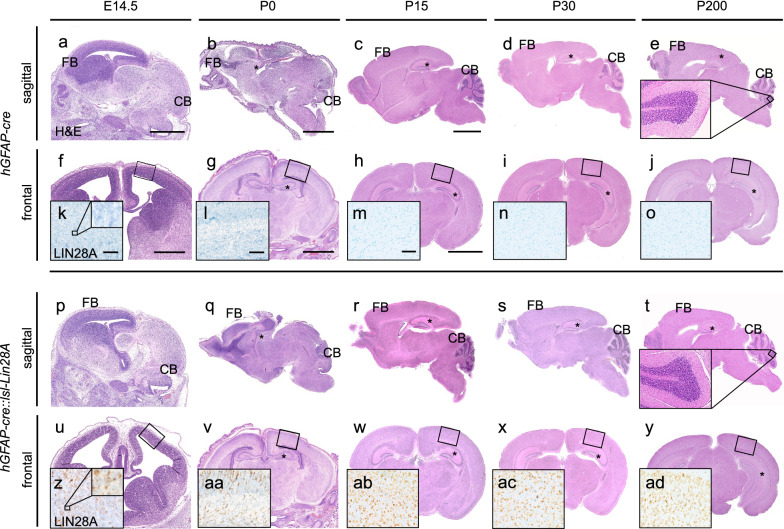
Overexpression of Lin28A in neural precursor cells does not lead to brain tumor formation. **a-j, p-y** H&E stained sagittal (**a-e**, **p–t**) and frontal (**f-j**, **u-y**) brain sections. Control (CTRL) (**a-o**) and GL (**p-ad**) mice did not display tumor formation in the forebrain or hindbrain on embryonic day 14.5 (E14.5), postnatal day 0 (P0), P15, P30 and up to P200. No abnormalities of foliation or cortical layering were detected in the cerebellum (insets in** e** and **t**). LIN28A stained mouse brains show a strong LIN28A expression in GL (**z-ad**) but not in CTRL mice (**k–o**). Scale bar in **a** is 1 mm for **a** and **p**; scale bar in **b** is 2.0 mm for **b** and **q**; scale bar in **c** is 2.5 mm for **c-e** and **r-t**; scale bar in **f** is 0.5 mm for **f** and **u**; scale bar in **g** is 2.0 mm for **g** and **v**; scale bar in **h** is 2.5 mm for **h-j** and **w-y**; scale bar in **k** is 50 µm for **k** and **z**; scale bar in **l** is 50 µm for **l** and **aa**; scale bar in **m** is 100 µm for **m–o** and **ab-ad**; FB = forebrain, CB = cerebellum, * = hippocampus

Early radial glia cells of the ventricular zone are proposed as cells of origin for ETMR [28]. In line with this, we previously demonstrated the formation of ETMR-like tumors by increasing Sonic hedgehog- and Wnt signaling in *hGFAP*-positive ventricular precursor cells of the mouse [46]. As ETMRs show an overexpression of Lin28A, we wanted to examine whether Lin28A is sufficient to drive embryonal brain tumors in vivo. Therefore, we overexpressed Lin28A in *hGFAP-cre::lsl-LIN28A* (GL) mice (Fig. 1, Additional File 1: Fig. 1a). Since the *hGFAP* promotor begins to drive Cre expression on embryonic day E13.5 [83], we analyzed the fore- and hindbrain of GL mice starting at embryonal day E14.5 up to postnatal day 200 (Fig. 1). We did not find any brain tumors in GL mice (Fig. 1p–y). In contrast to littermate controls (CTRL), GL mice displayed strong LIN28A expression (Fig. 1 z-ad). Fatemapping analyses using *hGFAP-cre::R26tdRFP*^*fl/*+^ mouse brains confirmed a strong expression of red fluorescent protein (RFP) within the forebrain, including the cortex and hippocampus and the hindbrain including the cerebellar cortex (Additional File 1: Fig. 1b-q). Compared to CTRL mice, GL mice did not significantly differ regarding body weight, brain weight or cortical brain thickness (Additional File 1: Fig. 1r-v). However, Kaplan Meier analyses showed a decreased survival of GL mice compared to CTRL mice with a proportion of GL animals dying between day 27 and 67 (*p* = 0.0028, log-rank test, n = 83 for CTRL, n = 58 for GL, Additional File 1: Fig. 1w). Therefore, we additionally analyzed other organs such as heart, kidney, spleen, intestine, liver, pancreas and lung (Additional File 1: Fig. 2a). While overexpression of Lin28A was detected in single cells of the intestine, liver, pancreas and lung of GL mice, we did not detect tumors or obvious histopathological changes in organ morphology (Additional File 1: Fig. b-aq). Of the 8 GL mice that died (Additional File 1: Fig. 1w), only four could be thoroughly histomorphologically evaluated as the quality and availability of tissue was limited by extended post-mortem intervals. No tumor like lesions or significant morphological differences could be detected in comparison to inconspicuous GL mice. Additionally, differential blood counts of 15-week-old mice revealed slightly higher hemoglobin and hematocrit values in GL mice (*p* = 0.0286 and *p* = 0.0197, unpaired t-test, n = 4 for CTRL, n = 4 for GL, Additional File 1: Fig. 2 ar). Neutrophil granulocytes were decreased (p = 0.0286, unpaired t-test, n = 4 for CTRL, n = 4 for GL) and monocytes as well as lymphocytes were increased (p = 0.0129 and p = 0.0459, unpaired t-test, n = 4 for CTRL, n = 4 for GL, Additional File 1: Fig. 2 ar). Papanicolaou staining did not reveal any obvious cytomorphological changes of GL blood cells when compared to CTRL (Additional File 1: Fig. 2as, at).

In summary, GL mice showed decreased survival and slight changes of blood cell counts but no tumor formation was detected in the brain or other organs.

### Overexpression of Lin28A in GL mice results in transiently increased cell proliferation within the cerebral cortex.

**Fig. 2:**
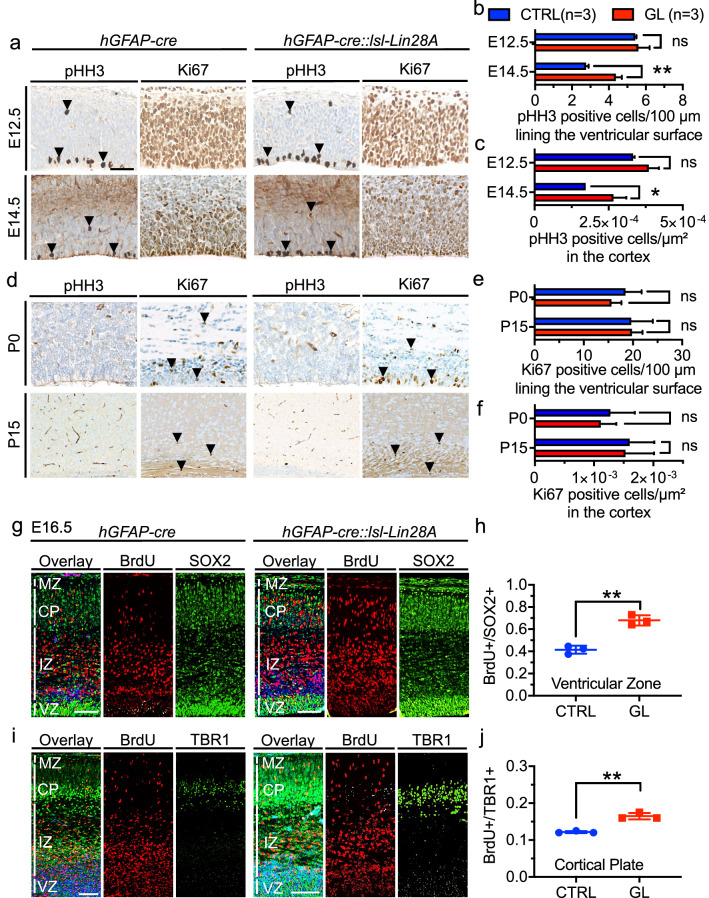
Overexpression of Lin28A results in transiently increased cell proliferation within the cerebral cortex. **a**, **d** PHH3 and Ki67 stainings of the cerebral cortex of CTRL and GL mice at different time points (E12.5, E14.5, P0 and P15). **b**, **c** Quantification of pHH3-positive cells. GL mice showed increased proliferation of cells lining the ventricles (**b**, p = 0.0014, unpaired t-test, n = 3 for CTRL, n = 3 for GL) and of cells in the whole cerebral cortex (**c**, p = 0.0212, unpaired t-test, n = 3 for CTRL, n = 3 for GL) when compared to CTRL mice at E14.5. **e**, **f** Quantification of Ki67-positive cells. No significant difference in proliferation at postnatal stages. **g-j** Double immunofluorescence staining of the cerebral cortex at E16.5 after BrdU application at E14.5. **g** BrdU and SOX2 co-staining for detection of BrdU positive cells in the ventricular zone (VZ). **h** In GL mice the fraction of BrdU + /SOX2 + cells of the VZ was significantly increased (*p* = 0.0014, unpaired t-test, n = 3 for CTRL, n = 3 for GL). **i** BrdU and TBR1 co-staining for detection of BrdU positive cells in the cortical plate (CP). (**j**) The fraction of BrdU + /TBR1 + cells in the cortical plate was significantly increased in GL mice (*p* = 0.0012, unpaired t-test, n = 3 for CTRL, n = 3 for GL). Scale bar in **a** is 50 µm for **a** and **d**; scale bars in **g** and **i** are 100 µm; arrows exemplary mark pHH3 and Ki67 positive cells. VZ = ventricular zone, IZ = intermediate zone, CP = cortical plate, MZ = marginal zone. CTRL n = 3, GL n = 3 for **i**, **r**, **y**, **ff**. P values are shown in figures as ns = not significant (*p* > 0.05), *p < 0.05, ** p < 0.01, ***p < 0.001, ****p < 0.0001

As Lin28A has been shown to be associated with brain tumors and neural progenitor proliferation [33, 46, 75], we next aimed to analyze the influence of Lin28A overexpression on cell proliferation within the cerebral cortex. Quantification of pHH3-positive cells (indicating mitotic cells) showed increased numbers of positively stained cells at the ventricular surface and within the cerebral cortex at E14.5 in GL mice when compared to CTRL mice (*p* = 0.0014 and *p* = 0.0212, unpaired t-test, n = 3 for CTRL, n = 3 for GL, Fig. 2a–c). As only scarce pHH3-positive cells were detected at postnatal stages, Ki67 staining (proliferation marker) was used for quantification of timepoints P0 and P15. No significant differences of Ki67-positive cell numbers were detected in the brain cortex at P0 and P15 (Fig. 2 d-f). To confirm the results of pHH3 staining and follow the fate of embryonal proliferating cells, we applied BrdU to pregnant female mice at E14.5 and analyzed the offspring’s brain cortex at E16.5. Double immunofluorescence staining was applied to label the fraction of BrdU-positive cells among SOX2-positive progenitor cells of the cortical ventricular zone (VZ). GL mice displayed a significantly increased fraction of BrdU + /SOX2 + cells in the VZ (p = 0.0014, unpaired t-test, n = 3 for CTRL, n = 3 for GL, Fig. 2 g, h). BrdU and TBR1 co-staining for detection of BrdU positive cells in the cortical plate (CP) further showed a significantly increased fraction of BrdU + /TBR1 + cells in the cortical plate in GL mice (p = 0.0012, unpaired t-test, n = 3 for CTRL, n = 3 for GL, Fig. 2 i, j). In order to investigate if Lin28A impacted on the distribution of mitotic phases, we analyzed the morphology of cell divisions in the cortex of embryonal GL mice but did not detect significant differences compared to CTRL (E14.5, each n = 4, p = 0.6489, chi square, Additional File 1: Fig. 3a, b). Moreover, we investigated the mitotic spindle orientation at the ventricular lining, a feature being linked to asymmetric and symmetric cell divisions [43]. We detected a trend towards increased vertical cell divisions in GL mice, however, results were not significant (*p* = 0.3325, chi square, Additional File 1: Fig. 3c–e).

In conclusion, overexpression of Lin28A in GL mice resulted in transiently increased proliferative activity within the cerebral cortex during embryonal development, which was not maintained at postnatal stages.

### GL mice show LIN28A overexpression in neurons of the cerebral cortex and hippocampus.

**Fig. 3:**
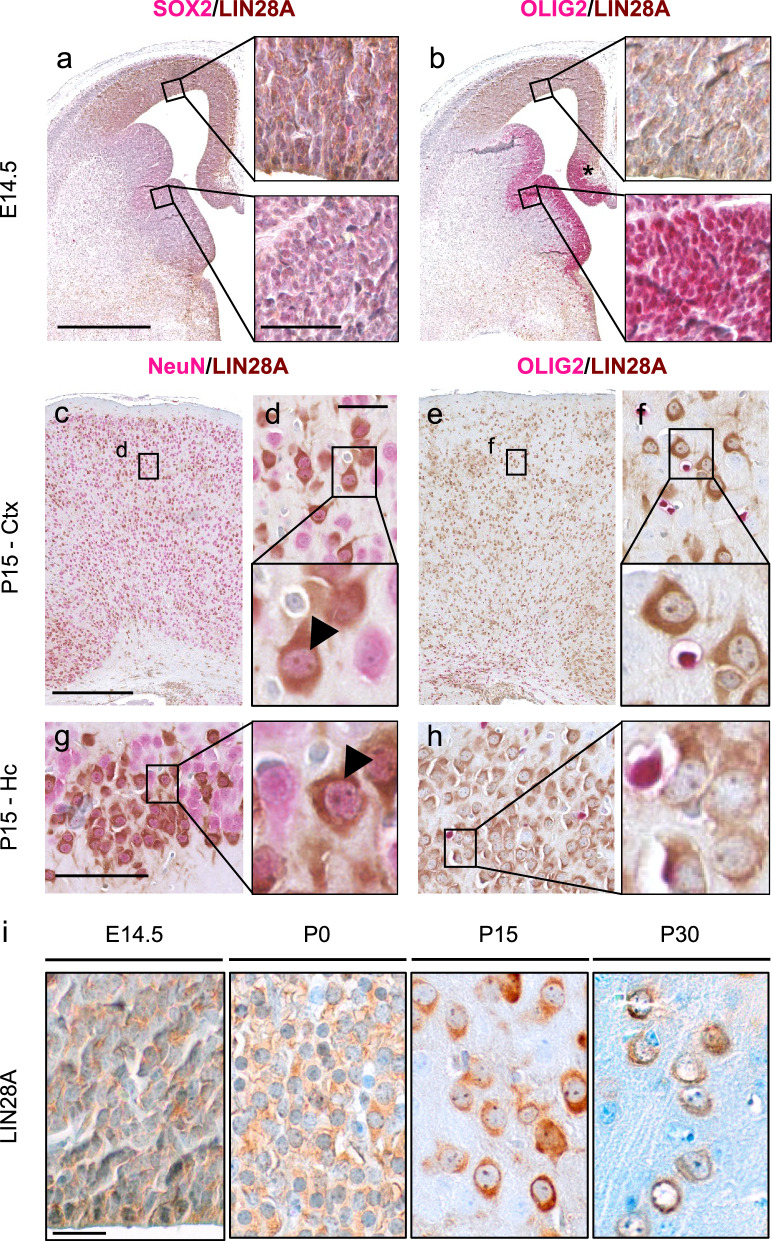
GL mice show LIN28A overexpression in neurons of the cortex and hippocampus. **a** Costainings of LIN28A (brown) and SOX2 (red) show LIN28A positivity in the SOX2-positive cortical ventrical zone. **b** Costainings of LIN28A (brown) and OLIG2 (red) show lack of LIN28A expression in the OLIG2-positive ventrical zone of the lateral and medial ganglionic eminences. Costaining is found in the region of the developing hippocampus (*). (**c**) NeuN-positive neurons in the cortex express LIN28A (**c**, **d**, arrowhead). No colocalization of LIN28A-positive cells and OLIG2-positive cells in the cerebral cortex at P15 (**e**, **f**). **g** NeuN-positive neurons in the pyramidal layer of the hippocampus strongly express LIN28A (arrowhead). No colocalization of LIN28A-positive cells and OLIG2-positive in the hippocampus (**h**). For investigation of subcellular expression patterns, high magnifications of LIN28A stained sections of GL brains are shown at different time points. At embryonal and postnatal stages, LIN28A was found in the cytoplasm (**i**). Scale bar in **a** is 500 μm for **a** and **b**; scale bar inset **a** is 50 μm for **a** and **b**; scale bar panel **c** is 500 μm for **c** and **e**; scale bar panel **d** is 50 μm for **d** and **f**; scale bar panel **g** is 100 μm for **g** and **h**; scale bar in **i** is 20 μm for all time points; Ctx = cortex, Hc = hippocampus

Having seen no tumor formation but changes in proliferation within the ventricular zone, we next asked which cell types were primarily targeted in the GL mouse model. Therefore, co-immunostainings of LIN28A with nuclear markers were performed at embryonal and postnatal stages (Fig. 3 a-h). Consistent with the activity of the *hGFAP-cre* promotor [46, 83], LIN28A expression was mainly found in the SOX2-positive embryonic ventricular zone of the cerebral cortex, but not in the OLIG-positive ventricular zone of the lateral and medial ganglionic eminences (E14.5, Fig. 3 a, b). At postnatal day 15, LIN28A was then found in NeuN-positive neurons of the cortex and hippocampus but not in OLIG2-positive glia cells (P15, Fig. 3 c-h). In line with its function as an mRNA binding protein [18], we detected a cytoplasmic staining pattern of LIN28A at embryonal and postnatal stages (Fig. 3 i).

In conclusion, GL mice displayed an overexpression of LIN28A in the cytoplasm of embryonal neural progenitors and postnatal neurons.

### Lower spine density in the cortex of GL mice.

**Fig. 4:**
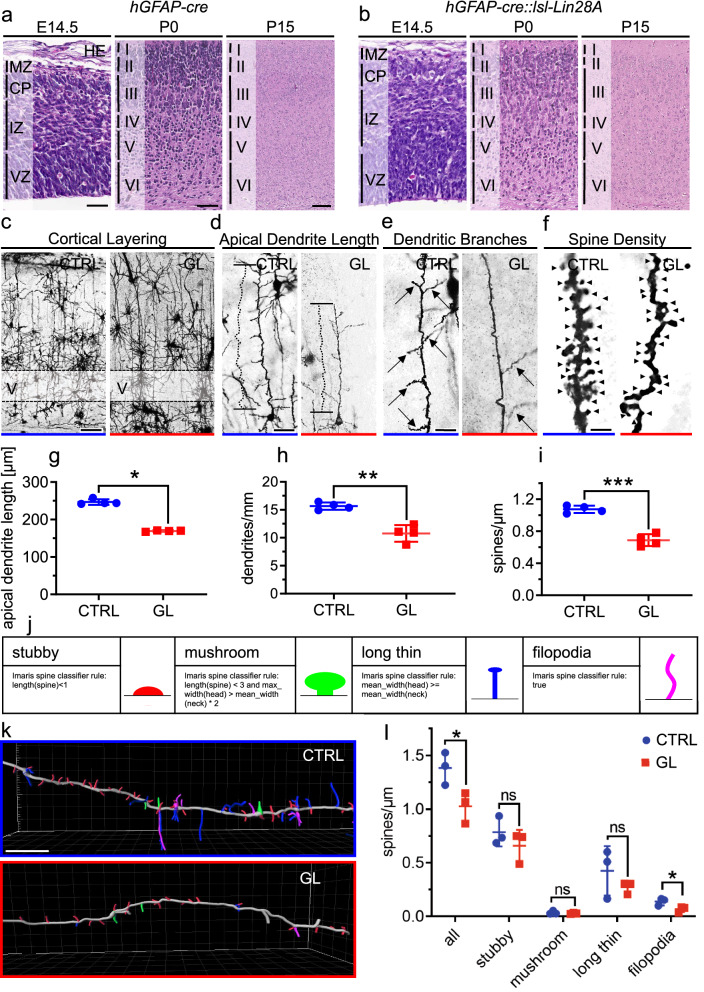
Lower spine density and dendritic alterations of cortical pyramidal layer V neurons in GL mice. **a, b** H&E staining of frontal sections of the cerebral cortex (P15). CTRL (**a**) and GL mice **b** displayed similar layering during development (E.14.5) and isocortical layering at postnatal stages (P0, P15). **c-f** Rapid Golgi Cox staining of the isocortex in CTRL and GL mice at P15. Representative images of cortical layering (**c**), layer V pyramidal cells’ apical dendrite length (**d**), dendritic branching (**e**) and spines of apical dendrite branches (**f**). **g** Decreased apical dendrite length in GL mice compared to CTRL (*p* = 0.0286, Mann–Whitney test, n = 4 for CTRL, n = 4 for GL). (**h**) Less dendritic branching in GL mice compared to CTRL (*p* = 0.0010, Mann–Whitney test, n = 4 for CTRL, n = 4 for GL). (**i**) Significant reduction of spines in GL mice (*p* = 0.0001, Mann–Whitney test, n = 4 for CTRL, n = 4 for GL). (**j**) Spine classification defined as stubby, mushroom, long thin and filopodia by Imaris and MATLAB R2018b rules. (**k**) Representative spine classification in DiI-stained apical dendrite branches of CTRL and GL mice. (**l**) Overall decreased spine density (p = 0.0424, t-test, n = 3 for CTRL, n = 3 for GL) and reduction of filopodia (p = 0.0468, t-test, n = 3 for CTRL, n = 3 for GL) in GL compared to CTRL. Scale bar in **a** is 50 µm for E.14.5; 50 µm for P0 and 50 µm for P15 for **a**, **b**; scale bar in **c** is 100 µm; scale bar in **d** is 50 µm; scale bar in **e** is 20 µm for scale bars in **f** and **k** are 5 µm. VZ = ventricular zone, IZ = intermediate zone, CP = cortical plate, MZ = marginal zone, I-VI = cortex layer I-VI. CTRL n = 4, GL n = 4 for **g**-**i**; CTRL n = 3, GL n = 3 for **l**. ns = not significant (*P* > 0.05). P values are shown in figures as *p* < 0.05, **p < 0.01, ***p < 0.001, ****p < 0.0001

Next, we compared cortical layering of GL mutants and CTRL mice in more detail. H&E staining revealed proper layering during embryonal development and isocortical layering at postnatal stages in CTRL and GL mice (Fig. 4 a, b). Specifically, a similar distribution of SOX2 positive cells in the VZ, TBR2-positive cells in the subventricular zone, TBR1-positive early born neurons (embryonal stages) and layer VI cortical neurons (postnatal stages), CTIP2-positive layer V cortical neurons and Reelin-positive Cajal-Retzius in the marginal zone layer of both in CTRL and GL mice were seen (Additional File 1: Fig. 4). We therefore concluded that Lin28A overexpression did not significantly affect cell migration and fate during cortical development.

Since we have seen a clear colocalization of LIN28A and NeuN in GL mice, we further analyzed neuron and spine morphology using Rapid Golgi Cox staining (Fig. 4 c-i). Apical dendrite length, dendritic branching and spines of apical dendrite branches were quantified in layer V pyramidal cells (Fig. 4 c-f). Compared to CTRL, GL mice showed decreased apical dendrite length (*p* = 0.0286, Mann–Whitney test, n = 4 for CTRL, n = 4 for GL), less dendritic branching (p = 0.0010, Mann–Whitney test, n = 4 for CTRL, n = 4 for GL) and a significant reduction of spines (p = 0.0001, Mann–Whitney test, n = 4 for CTRL, n = 4 for GL, Fig. 4 g-i). In order to confirm these results and to further analyze dendritic spine morphology, we used DiI staining and classified spines as stubby, mushroom, long thin and filopodia by Imaris and MATLAB R2018b rules (Fig. 4 j). DiI stained apical dendrite branches of GL mice displayed an overall decreased spine density (p = 0.0424, unpaired t-test, n = 3 for CTRL, n = 3 for GL) with pronounced reduction of filopodia spines compared to CTRL (p = 0.0468, unpaired t-test, n = 3 for CTRL, n = 3 for GL, Fig. 4 k, l).

Taken together, Lin28A overexpression in neural precursors did not alter cortical layering but resulted in a significant decrease of dendritic spine density in layer V pyramidal cells.

### Pyramidal cell layer dispersion and reduced number of spines in the hippocampus of GL mice.

**Fig. 5:**
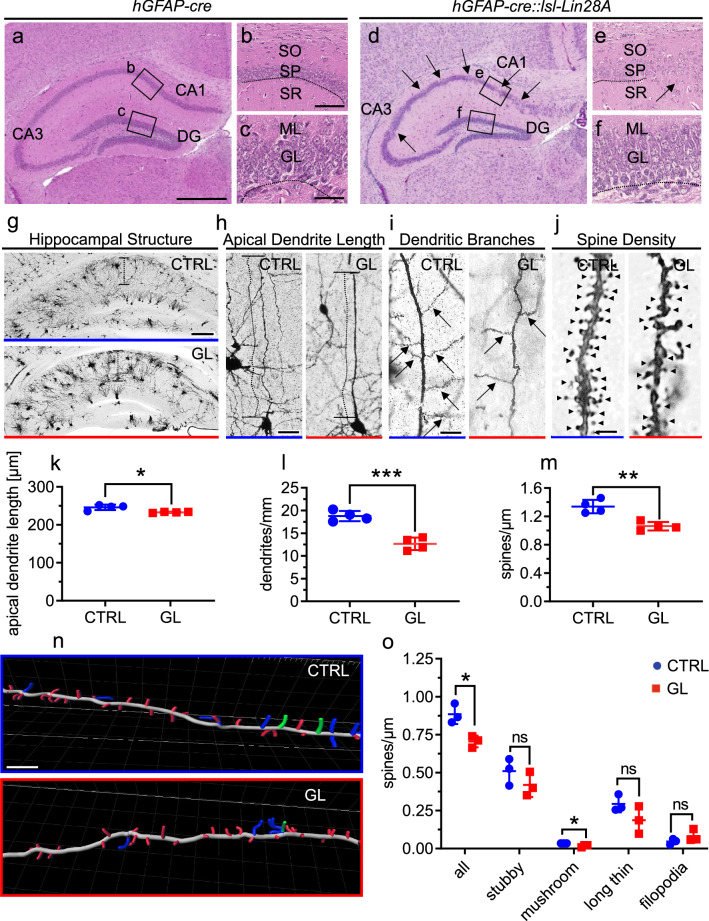
Lower spine density, neuronal alterations and pyramidal cell dispersion in the hippocampus of GL mice. (**a-f**) H&E staining (P15) of frontal hippocampal sections of CTRL (**a-c**) and GL mice (**d-f**). **d**, **e** Pyramidal cell dispersion in CA1—CA3 regions of the hippocampus in GL mice compared to CTRL. Lines emphasize smooth layering, arrows in **d** and **e** mark areas of cell dispersion. (**g-j**) Rapid Golgi Cox staining of the hippocampus for CTRL and GL mice. Representative images of CA1-CA3 hippocampal structure (**g**), hippocampal pyramidal cell’s apical dendrite length (**h**), dendritic branching (**i**) and spines of apical dendrite branches (**j**). Decreased apical dendrite length (p = 0.0286, Mann–Whitney test, n = 4 for CTRL, n = 4 for GL) (**k**), less dendritic branching (p = 0.0005, Mann–Whitney test, n = 4 for CTRL, n = 4 for GL) (**l**) and significantly reduced spines (*p* = 0.0026, Mann–Whitney test, n = 4 for CTRL, n = 4 for GL) (**m**) in GL mice. **n** Representative spine classification in DiI-stained apical dendrite branches of CTRL and GL mice. **o** Overall decreased spine density (p = 0.0143, t-test, n = 3 for CTRL, n = 3 for GL) with reduction of mushroom spines (p = 0.0170, t-test, n = 3 for CTRL, n = 3 for GL) in GL mice compared to CTRL. Scale bar in **a** is 200 µm for **a** and **d**; scale bar in **b** is 100 µm for **b** and **e**; scale bar in **c** is 100 µm for **c** and **f**; scale bar in **g** is 200 µm; scale bar in **h** is 50 µm; scale bar in **i** is 20 µm; scale bars in **j** and **n** are 5 µm. CA = cornu ammonis, DG = dentate gyrus; SO = stratum oriens hippocampi, SP= stratum pyramidale hippocampi, SR = stratum radiatum hippocampi, ML = molecular layer, GL = granular cell layer. CTRL n = 4, GL n = 4 for **k-m**; CTRL n = 3, GL n = 3 for **o**. ns = not significant (*p* > 0.05), *p < 0.05, **p < 0.01, *** *p* < 0.001, ****p < 0.0001

Next, we assessed the hippocampal architecture of GL mice. H&E staining showed a granule cell dispersion of the hippocampus involving CA1 and 3 regions in GL mice which we did not see in CTRL mice on P15 (Fig. 5 a-f). In order to analyze CA region specificity, we stained the CA1 region marker WFS and the CA3 region marker HUB but did not detect a difference of staining patterns (Additional File 1: Fig. 5 a-l). Further immunostaining analyses confirmed LIN28A positive cells in the pyramidal layer and dentate gyrus (DG) of GL mice but no major differences of cells expressing NeuN, REEL, CTIP2, TBR2, or SOX2 were detected when comparing CA1 and DG regions of GL and CTRL hippocampi (Additional File 1: Fig. 5 m-aj). Additionally, Ki67 stainings did not reveal significant differences of proliferating cells of the subgranular zone (Additional File 1: Fig. 5 ak, al, am, an). To further analyze neuron and spine morphology of hippocampal pyramidal cells, we again applied Rapid Golgi Cox staining (Fig. 5 g-j). We observed decreased apical dendrite length (p = 0.0286, Mann–Whitney test, n = 4 for CTRL, n = 4 for GL), less dendritic branching (p = 0.0005, Mann–Whitney test, n = 4 for CTRL, n = 4 for GL) and significantly reduced spines in GL mice compared to CTRL (p = 0.0026, Mann–Whitney test, n = 4 for CTRL, n = 4 for GL, Fig. 5 k-m). Following up, DiI staining was used to classify spine morphologies (Fig. 5 n, o). DiI stained apical dendrite branches of GL mice displayed an overall decreased spine density (p = 0.0143, unpaired t-test, n = 3 for CTRL, n = 3 for GL) with pronounced reduction of mushroom spines compared to CTRL (p = 0.0170, unpaired t-test, n = 3 for CTRL, n = 3 for GL, Fig. 5 o). In conclusion, Lin28A overexpression resulted in a dispersion of the pyramidal cell layer of the hippocampus and a significant decrease of dendritic spines of hippocampal pyramidal cells.

As alterations in dendritic spines are associated with behavioral changes [17], we performed a modified open field test of GL and CTRL mice and analyzed kinetic activity with the animal tracking software ToxTrac [51] (Additional File 1: Fig. 6). We measured significant higher values for the parameters distance travelled (p = 0.0054, unpaired t-test, n = 12 for CTRL, n = 10 for GL), average speed (p = 0.0042, unpaired t-test, n = 12 for CTRL, n = 10 for GL) and average acceleration (p = 0.0099, unpaired t-test, n = 12 for CTRL, n = 10 for GL), in GL mice when compared to CTRL (Additional File 1: Fig. 6c-e). Anxiety parameters such as exploration rate, time spent in center and vertical activity did not reveal significant differences (Additional File 1: Fig. 6f–h).

To conclude, an overexpression of Lin28A in the hippocampus induced a pyramidal cell dispersion and resulted in a decreased dendritic spine density of pyramidal neurons. Concomitantly, GL mice demonstrated increased kinetic activity.

### Altered protein expression in brains of GL mice.

**Fig. 6:**
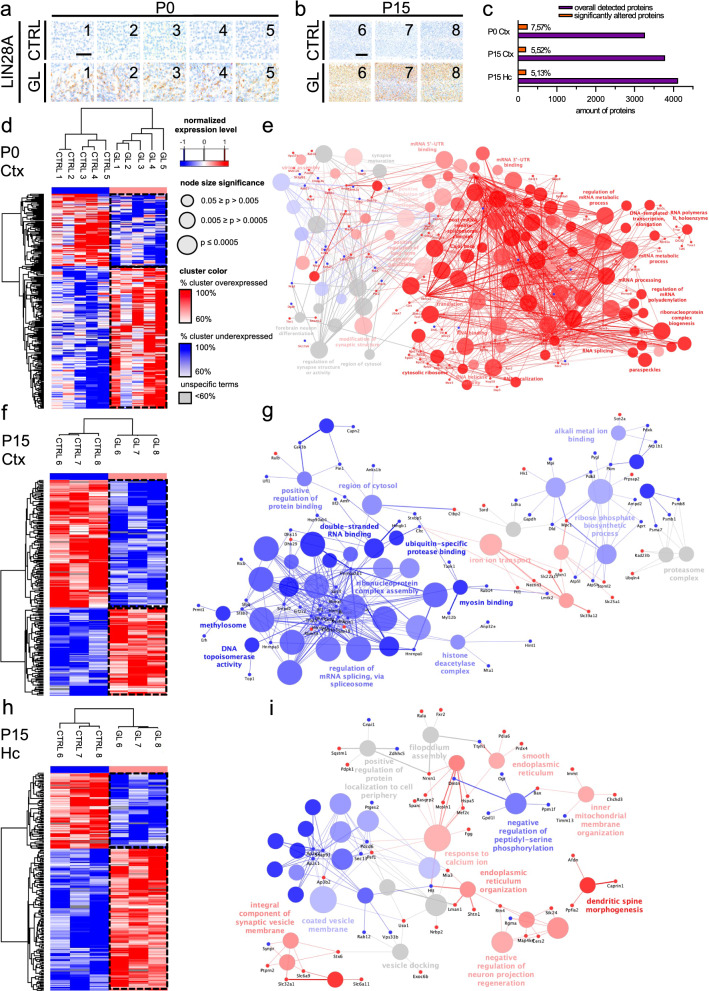
Altered protein expression in the cortex and hippocampus of GL mice. **a**, **b** LIN28A immunostaining of GL and CTRL brains used for proteome analyses at P0 (**a**) and P15 (**b**). **c** Significant altered proteins (with fraction) and overall detected proteins for P0 cortex (Ctx), P15 Ctx and P15 hippocampus (Hc). **d** Hierarchical clustering of significantly differentially abundant proteins in P0 GL cortices when compared to CTRL. **e** Specific gene ontology (GO) terms showed the top terms “regulation of synapse structure or activity” and “synapse maturation” for unspecific proteins and “RNA binding” and “mRNA processing” for overexpressed proteins. **f** Hierarchical clustering of significantly differentially abundant proteins in P15 GL cortices when compared to CTRL. **g** Specific GO terms showed the top terms “regulation of mRNA splicing, via spliceosome” and “ribose phosphate biosynthetic process” for decreased protein abundances and “iron ion transport” for increased protein abundances. **h** Hierarchical clustering of significantly differentially abundant in P15 GL hippocampi when compared to CTRL. **i** Specific GO terms showed the top terms “coated vesicle membrane” and “negative regulation of peptidyl-serine phosphorylation” for decreased protein abundances and “response to calcium ion” and “negative regulation of neuron projection regeneration “ for increased protein abundances. Scale bar in **d** is for **d**, **f** and **h**. Scale bar of node size significance is for **e**, **g** and **I**; scale bar of cluster color (shows the proportion of genes from each cluster that are associated to the term) in **e** (for **e**, **g** and **i**) is red for increased terms (100%-60%); scale bar of cluster color is blue for decreased terms (100%-60%). A term was called specific with > 60% genes being part of respective cluster, scale bar of cluster color is grey for unspecific terms (< 60%). Scale bar in **a** and **b** is 50 µm. CTRL n = 5, GL n = 5 for **d**, CTRL n = 3, GL n = 3 for **f** and **h**. P values as shown in GO term description

To unravel underlying molecular alterations in GL mice, we used western blot analyses and mass spectrometry (MS) based proteomics to analyze the global proteome of GL and CTRL brains (Fig. 6, Additional File 1: Fig. 7). All analyzed GL samples were confirmed to overexpress LIN28A in comparison to included CTRL samples based on immunohistochemistry and western blot (Fig. 6a, b, Additional File 1: Fig. 7). Human ETMR as well as our previous published ETMR mouse model display a strong WNT and SHH activation [46]. However, in line with the lack of tumor formation in the brains of GL mice, we did not observe a significant increase in the abundance of selected Wnt and Shh targets (APC, GLI2, AXIN2, MYCN, MYC, Additional File 1: Fig. 7a,b). Using mass spectrometry (MS) based proteomics, between 5.13% and 7.57% significantly altered proteins were detected in GL cortices at P0 and P15 and the GL hippocampus at P15 (Fig. 6 c).

A Gene ontology (GO) network displayed major terms involved in RNA transcription, translation and processing which could clearly be attributed to significantly increased protein abundance in GL cortex compared to control at P0 (Fig. 6 d, e). At P15, GL cortices showed similar GO terms related to transcription and translation which were, in contrast, attributed to significantly lower abundant proteins (Fig. 6 f, g). In the hippocampus of GL mice at P15 significantly differentially abundant proteins were assigned to GO-terms related to neuronal compartments and morphogenesis (Fig. 6 h, i). Detailed analyses of individual GO terms for each time point, with separated analyses for biological processes, KEGG & Reactome pathways and molecular functions & cellular components can be found in Additional file 2: Suppl. Table 1.

Taken together, MS-based proteomics showed altered molecular and functional processes in brains of GL mice mainly affecting RNA processing and neuron projection. It is remarkable that RNA processing proteins were increased in abundance at P0 but decreased in abundance at P15 in cortical GL tissue demonstrating a time dependency of Lin28A function.

### Phosphoproteomic analyses reveal downregulation of processes involving microtubule dynamics in GL mice.

**Fig. 7:**
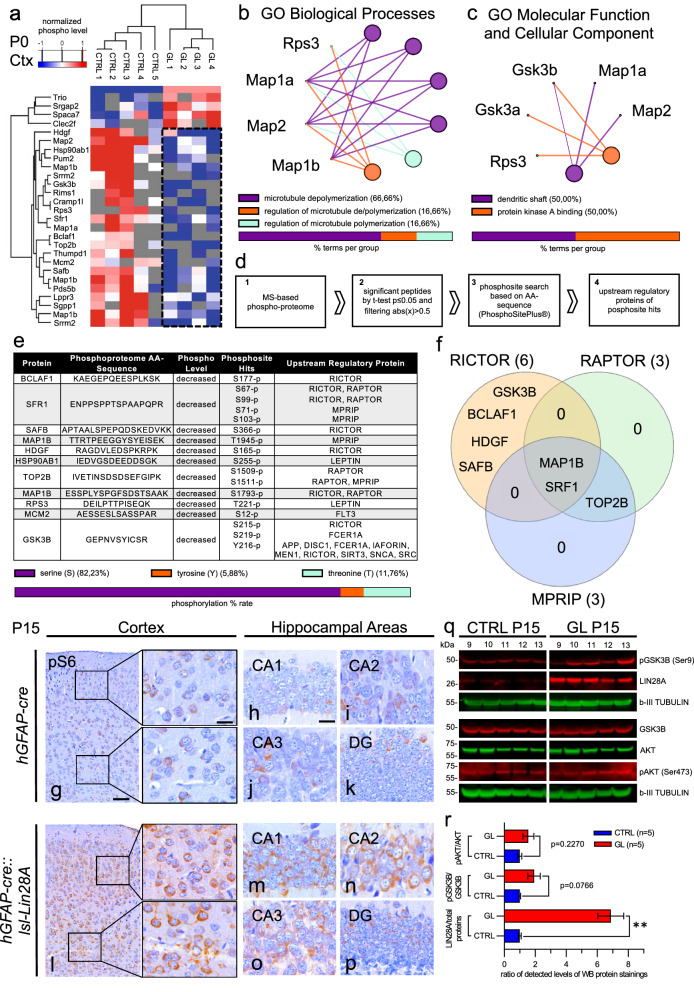
Phosphoproteomic analyses of GL mice. **a-c** MS-based phosphoproteomics of CTRL (n = 5) and GL cortices (n = 4) at P0. Hierarchical clustering of proteins showing significantly altered phosphosite abundance (decreased peptide phosphorylation levels in blue marked with dotted square) (**a**). Network analysis of GO Term Biological Processes based on decreased peptide phosphorylation levels displayed downregulated proteins of processes involved in microtubule dynamics (**b**). Network analysis of GO Term Molecular Functions and Cellular Component delivered the terms “dendritic shaft” and “protein kinase A binding” (**c**). **d-f** Detailed phosphosite analysis with upstream regulatory proteins and workflow (**d**). Decreased phosphosites were 82.35% serine sites, 5.88% tyrosine sites and 11.75% threonine sites (**e**). Venn diagram showing the top three upstream regulatory proteins RICTOR, RAPTOR and MPRIP regarding to phosphosite hits (**f**). **g-p** IHC staining of mTORC1 downstream signaling target Ser235/236 pS6 shows a stronger signal of positive cells in the cortex (**l**) and hippocampus (**m-p**) of GL mice when compared to CTRL mice (**g**, and **h–k**) at P15. **q**, **r** Western blot and quantification of pGSK3B, LIN28A, b-III TUBULIN, GSK3B, AKT and pAKT in cortices of GL and CTRL mice at P15 (every sample number represents an individual mouse). Significantly increased LIN28A (*p* = 0.0002, unpaired t-test, n = 5 for CTRL, n = 5 for GL) protein expression in GL mice compared to CTRL. No significant changes in pGKS3B/total GSK3B (*p* = 0.0766, unpaired t-test, n = 5 for CTRL, n = 5 for GL) and pAKT/total AKT ratios (*p* = 0.2270, unpaired t-test, n = 5 for CTRL, n = 5 for GL) in GL mice compared to CTRL (**r**). Scale bar in **g** is 100 µm for **l**; scale bar in insert of **g** is 25 µm for all inserts in **g** and **l**; scale bar in **h** is 250 µm for **h–k**, **m-p**. CA = cornu ammonis, DG = dentate gyrus

In order to gain further insights into pathway alterations and posttranslational modifications of proteins, we performed MS-based phosphoproteomics of GL (n = 4) and CTRL (n = 5) cortices at P0. A hierarchical clustering of proteins showing the top hits of significantly altered phosphosite abundance in GL mice versus CTRL mice is illustrated in Fig. 7 a. Phosphosites with decreased peptide phosphorylation levels were attributed to processes being involved in microtubule dynamics (Fig. 7 b, GO terms for biological processes). Network analysis of GO terms “Molecular Functions and Cellular Component” delivered the terms “dendritic shaft” and “protein kinase A binding” (Fig. 7 c). Detailed phosphosite analysis to uncover upstream regulatory proteins showed that phosphorylation distribution occurred 82.35% on serine sites, 5.88% tyrosine sites and 11.75% threonine sites (Fig. 7 d, e). Based on altered protein phosphosites, the top three upstream regulatory proteins RICTOR, RAPTOR and MRIP were identified using the PhospoSitePlus® 6.5.8 database [25] and illustrated in a synoptical venn diagram (Fig. 7 f) [20]. As RICTOR and RAPTOR are components of the mTOR pathway and LIN28A has been described to enhance mTOR signaling in different contexts [39, 79], we further analyzed the mTORC1 downstream signaling target Ser235/236 pS6 using immunohistochemistry. GL mice showed a stronger pS6 signal of positive cells in cortex and hippocampus when compared to CTRL mice at P15 (Fig. 7 g-p). Western blot analyses and quantification of P15 animals again confirmed significantly higher LIN28A protein expression in GL mice compared to CTRL (p = 0.0002, unpaired t-test, n = 5 for CTRL, n = 5 for GL, Fig. 7 q, r). The mTOR target Akt, being part of the PI3K/Akt pathway, together with its downstream target pGKS3B were not significantly altered in GL mice compared to CTRL mice though there was a tendency of increased pGKS3B/total GSK3B (p = 0.0766, unpaired t-test, n = 5 for CTRL, n = 5 for GL) and pAKT/total AKT ratios (p = 0.2270, unpaired t-test, n = 5 for CTRL, n = 5 for GL, Fig. 7 q, r).

In conclusion, phosphoproteomic analyses indicated a downregulation of mTOR pathway modulated proteins such as Map1b, which is involved in microtubule dynamics. The mTOR pathway associated members RICTOR and RAPTOR, together with MRIP were identified as possible upstream effectors.

Taken together, we show that Lin28A overexpression was not sufficient to drive brain tumors in vivo but impacted on spine morphogenesis. Our work provides new insights into the role of Lin28A during neuronal development and underlines that Lin28A functions are time and context dependent*.*

## Discussion

### Constitutive overexpression of Lin28A is not sufficient for brain tumor development

Lin28A is expressed during early embryonal development and in embryonal brain tumors, such as ETMR. We therefore constitutively overexpressed Lin28A in *hGFAP*-positive neural precursor cells to study its impact on brain development and for brain tumor formation in vivo. While *hGFAP*-positive precursor cells in the ventricular zone have been shown as a potential cell of origin for ETMR [46], a sole constitutive overexpression of Lin28A in these cells was not sufficient to drive brain tumor development, neither in the forebrain nor hindbrain. In line with these findings, Lin28A alone was not sufficient to constantly activate Shh and Wnt signaling in vivo. Still, Lin28A increased the mitotic rate of neural progenitors at embryonal stages. This indicates that Lin28A may contribute to malignant progression of tumor cells but additional factors are necessary to drive ETMR tumor formation in neural precursor cells. Whereas, an overexpression of LIN28A is a striking hallmark of human ETMR [34], additional specific alterations have been described such as an amplification of the miRNA cluster C19MC (Chr19q13.41) [32, 56]. Recent studies expect a C19MC-Lin28A-MYC circuit being responsible for the oncogenic potential in these tumors [56]. Noteworthy, the C19MC miRNA cluster is primate specific, which limits ETMR mouse model generation. The fact that LIN28A overexpression did not result in tumor formation does hence not preclude a potential role of LIN28A as a tumor-promoting factor in combination with other aberrations. Therefore, understanding the complexity of Lin28A function will be important to reveal future therapeutic options for ETMR patients.

### LIN28A expression accelerates neural progenitor proliferation during embryonal development

When investigating proliferation, a fundamental keystone in cancer promotion, we observed transient higher proliferation during embryonic developmental stages but similar proliferation levels in postnatal GL mice compared to CTRL mice. This is in line with previous findings, stating an important role for neural progenitor cell proliferation during embryonal development [22, 75]. Previous studies with sheep trophectoderm showed that overexpressed Lin28A/B decreased *let-7* miRNAs and increased IGF2BP1-3, HMGA1 and c-MYC, commonly known as genes, that are involved in cell proliferation [1]. In addition, exogenous LIN28A contributed to proliferation of neural progenitor cells in vitro and exerted a prosurvival effect on primary cortical neurons, possibly via upregulation of IGF-2 [7]. Interestingly, overexpression of the Lin28A homologue Lin28B resulted in a primarily cerebellar phenotype with prolonged granule neuron progenitor proliferation and hyperlobulation in a *Nestin-Cre::lsl-Lin28b* mouse model [66]. We did not detect such cerebellar alterations in our mouse model, indicating spatially distinct functions of both homologues. Despite lack of tumor formation in the brain or other organs, we observed a decreased survival rate of GL mice. We did not detect conspicuous macroscopic or histological abnormalities in other organs that could explain increased death rates, nor did mice show differences in weight or any preceding symptoms. Subtle changes in the differential blood cell count could be detected. While some reports relate Lin28A and its homologue Lin28B to hematopoiesis and also acute myeloid leukemia [14, 15, 80], it remains unclear if differences in blood counts are direct or indirect effects of Lin28A expression in *hGFAP*-positive cells. One might speculate that hippocampal pyramidal cell dispersion within the CA1 and CA3 regions of GL mice might explain significant death events. As part of mesio-temporal lope epilepsy (MTLE) and epilepsy, loss of pyramidal neurons, especially within CA1 and CA3, was correlated with cytoskeletal abnormalities [10, 59, 77]. Furthermore, proteome analyses of patients with MTLE uncovered an elevated expression of Rho GTPases [36] and, interestingly, also showed an increase of mTOR downstream target pS6 in the hippocampus [54]. We did not detect any differences in clinical behavior between short time and longtime survivors (e.g. both showed hyperactive phenotypes). Both male and female mice deceased and there were no clinical signs prior to a potential death event during monitoring. In the context of increased kinetic activity “functional abnormalities” such as epileptic seizures are worth to be considered as a potential cause of death in GL mice – these events might occur suddenly without previous symptoms and might also not be detected by histological assessment. Of note, the mTOR pathway which was found to be altered in GL mice has been linked to epilepsy phenotypes in humans [23, 41, 73].

### Lin28A overexpression may lead to hyperkinetic behavioral disorders

GL mice showed a pyramidal cell dispersion in the hippocampus and decreased spine densities in the cortex and hippocampus. Additionally, we could explore a more active phenotype in an open field test when analyzing parameters such as distance travelled, average speed and average acceleration. These findings suggest hyperkinetic behavior in Lin28A overexpressing mice. Interestingly, previous studies described a *Nestin-Cre/Lin28a/b-cKO* knockout mouse model that showed a degeneration of midbrain type dopamine neurons (mDA) in the substantia nigra. In contact with MPTP toxin, this was accompanied by a phenotype related to Parkinson’s Disease (PD) with hypokinetic movement [13, 74]. Intriguingly, a loss-of-function mutation of *LIN28A* (R192G substitution) could also be observed in two early-onset PD patients within an Asian population [13]. The impact of LIN28A function on hypokinetic disorders should therefore be of further interest for future studies.

### Lin28A function is time, cell and context dependent and influences microtubule dynamics in a crosstalk between m-TOR and Gsk3b/Rho-Rac/Map1b signaling

Proteomics and network analysis of GL mice at P0 showed an overrepresentation of proteins involved in transcriptional and translational processes. This is well in line with the literature, where Lin28A has been reported to bind mRNAs, regulate splicing and translational processes in stem cells [49, 70]. Additionally, Lin28A has been described to act in the nucleus being involved in transcriptional processes [5, 31]. In contrast, our analyses of P15 cortical tissue of GL mice showed that mRNA regulation mechanisms were underrepresented in GL mice in comparison to CTRL. One might hypothesize that an overexpression of Lin28A shifts developmental processes with increased proliferation and translational/transcriptional processes at earlier time points (until P0) which then decrease more quickly in comparison to CTRL at later time points (as detected on P15). A dysregulated timing in the development of progenitor cells and differentiation of neurons and glia cells after constitutive Lin28A overexpression has also been proposed before [30, 52]. Moreover, recent studies showed, that Lin28A leads to a general higher neuronal turnover while the RNA-silencing factor TRBP promotes Lin28A stabilization [2]. Supplementary, secondary mechanisms might influence effects of a constitutive Lin28A expression in a time-and context specific manner.

Additionally, proteome analyses of GL mice identified GO-terms related to neuronal compartments and morphogenesis at P15, matching to a decrease in spine density seen in Golgi and DiI stainings. Lin28A has recently been described to be involved in synaptic plasticity [26] and LIN28A resulted in reduced neuronal synapse density in vitro using iPSC lines [30].

We could identify the mTOR pathway components Rictor and Raptor, as well as Gsk3b and Map1b as potential key regulators in the phosphoproteomic analyses of GL mice. Interestingly, the mTOR pathway has also been related to Parkinson’s Disease [68, 82] whereas autophagy regulation had an impact on social behavior in autism spectrum disease mouse models with hyperactive mTOR [61]. Physiologically, degradation of Rictor inhibits AKT activation which again leads to a higher Gsk3b activity [71]. This regulates axonal outgrowth and axon formation in neurons via calcium influx in axonal growth cones [65]. Opposed to this, we could identify upregulated mTOR pathway activity via pS6 staining, tendentially higher pAKT and lower Gsk3b ratios in GL mice. Intriguingly, Gsk3b deletion in cortical and hippocampal neurons of adult mice also results in reduced spine density [47]. Phosphorylation on the S9-p site of GSK3B leads to inhibition of Gsk3b activity, which modulates the association of APC with the plus-end of microtubules [29]. We also found a tendentially higher pGsk3b/Gsk3b ratio on the S9-p site using western blotting and quantitative differential proteomics showed biological processes attributed to microtubule dynamics such as “microtubule de/polymerization”.

We also identified decreased phosphorylation levels of the microtubule associated protein Map1b. Map1b deficient mice show decreased spine density, and Map1 deficient cultured neurons display a delay in axon outgrowth and elongation [42, 62]. Hereby, a significant decrease in Rac1 and increase of Rho activity could be observed, suggesting a crosstalk between microtubules and actin cytoskeleton in neuronal polarization via Rho-Rac signaling [42]. In phosphoproteomic analyses of GL mice we could observe higher phosphorylation levels of TRIO, which is a Guanine nucleotide exchange factor (GEF) for RHOA and RAC1 GTPases [6], and the related upstream regulatory protein MPRIP (Myosin phosphatase Rho-interacting protein) [45]. Interestingly, MPRIP was shown to be essential for RhoA/ROCK-regulated neuritogenesis by influencing the actin cytosceleton [45]. Thus, it seems that Lin28A is involved in the regulation of microtubule dynamics, playing an essential role in dendritic spine morphology [27]. Future experiments will show the precise role of how LIN28A controls microtubule dynamics.

Taken together, our results show that Lin28A overexpression in vivo is not sufficient to drive brain tumors but leads to a reduced spine density in GL mice with underlying molecular alterations being a complex signaling crosstalk between m-TOR pathway and Gsk3b/Rho-Rac/Map1b signaling affecting microtubule dynamics.

## Methods

### Transgenic mouse models

*hGFAP-cre* mice [83] and *lsl-R26tdRFP*^*fl/fl*^ mice [38] were obtained from The Jackson Laboratories (Bar Harbor, ME, USA). Cre inducable *lsl-Lin28A* mice and the *LIN28A(3x)-IRES-eGFP* insert have been published before [46, 48]. Mice were kept on a 12 h dark/light cycle and had open access to water and food. Both male and female mice were examined. Control (CTRL) mice were littermates of *hGFAP-cre:: lsl-Lin28A* (GL) mice harbouring exclusively either the *hGFAP-cre or lsl-Lin28A* transgene*.*

All experiments using animals were approved by the local animal care committee (Behörde für Lebensmittelsicherheit und Veterinärwesen in Hamburg) and handling was conducted in accordance with local governmental and institutional animal care regulations.

### Genotyping

DNA was extracted from ear or tail biopsies using Laird’s buffer (10 mM Tris–HCl pH 8.5, 5 mM EDTA, 0.5% SDS, 0.2 M NaCl, 0,1 mg/ml protein kinase K in ddH2O) and Isopropanol precipitation. DNA was dissolved in DEPC water (0.1% DEPC dissolved in ddH2O and autoclaved) and stored at 4 °C. Genotype-specific regions of the genome were amplified via PCR utilizing the following primers (FW 5’-3’, REV 5’-3’) (Cre: TCCGGGCTGCCACGACCAA, GGCGCGGCAACACCATTTT; RFP: AAAGTCGCTCTGAGTTGTTAT, GGAGCGGGAGAAATGGATATG; lsl-Lin28A:

ATCTTATCATGTCTGGAT CCCC, CGCAGTTGTAGCACCTGTCTC) and a DreamTaq-Polymerase (Thermo Scientific, EP0703) based standard reaction mixture.

### BrdU Assay

For in vivo analysis of proliferation and migration, mice were pulse-labeled with BrdU (5-bromo-2-deoxyuridine) (Sigma, B5002-100MG) at a concentration of 50 mg/kg body weight that was applied i.p. to pregnant female mice on E14.5. Embryos were sacrificed two days later on E16.5.

### Differential blood counts

For hematology blood analysis, 70 µl blood was collected from the submandibular vein using heparinized micro-hematocrit tubes (Vitrex, Product No. 161813). Afterwards, Blood was saved in tubes with prefilled EDTA (Kabe Labortechnik GmbH, Product No. 077011). Then, using a ProCyte Dx Hematology Analyzer (IDEXX Laboratories), differential blood counts were analyzed from EDTA-anticoagulated whole blood samples within 4 h after blood withdrawal. Using optical fluorescence, laminar flow impedance, and cyanide-free sodium lauryl sulphate (SLS)—hemoglobin method, determination of the represented parameters were obtained in one run [58].

### Immunohistochemistry (IHC)

For hematoxylin and eosin (H&E) and immunohistochemical (IHC) staining, tissues were fixed with 4% buffered formalin for at least 24 h at room temperature and processed for paraffin embedding. After embedding in paraffin using a Leica ASP300S tissue processor with a Leica EG1160 embedding station (Leica Microsystems CMS GmbH, Wetzlar, Germany), Sects. (2 µm) were subjected to H&E staining or processed for immunohistochemistry as follows: After dewaxing and inactivation of endogenous peroxidases (PBS/3% hydrogen peroxide), antibody specific antigen retrieval was performed using the Ventana Benchmark XT machine (Ventana, Tuscon, USA). Sections were blocked (PBS/10% FCS) and afterwards incubated with the following primary antibodies: mouse anti-BrdU (abcam, ab131137, 1:100), rat anti-Ctip2 (abcam, ab18465, 1:50), rabbit anti-HuB (Sigma, H1538, 1:200), rabbit anti-Ki67 (abcam, ab15580, 1:100), rabbit anti-Lin28A (Cell Signaling Technology, 3978, 1:100), mouse anti-NeuN (Merck, MAB377, 1:100), mouse anti-Olig2 (Merck, MABN50, 1:100), mouse anti-pHH3 (Cell Signaling Technology, 9706L, 1:200), rabbit anti-pS6 (Cell Signaling, #4858, 1:200), mouse anti-Reelin (Merck, MAB5364, 1:100), rabbit anti-RFP (Antibodies online, ABIN129578, 1:50), rabbit anti-Sox2 (abcam, ab97959, 1:200) and rabbit anti-Tbr1 (abcam, ab31940, 1:50), rabbit anti-Tbr2 (abcam, ab23345, 1:500), rabbit anti-WFS1 (Proteintech, 11,558–1-AP, 1:50). Sections were incubated with primary antibody for 1 h, anti-rabbit or anti-mouse Histofine Simple Stain MAX PO Universal immunoperoxidase polymer (Nichirei Biosciences, Wedel, Germany) were used as secondary antibodies. Detection of secondary antibodies and counter staining was performed with an ultraview universal DAB detection kit from Ventana (Ventana, Tuscon, USA).

### Immunofluorescence (IF-P)

After dewaxing paraffin embedded brain slides with a descending alcohol series, antigen retrieval was performed with a citrate buffer (pH 6.0). Next, brain slides were briefly washed with PBS and then treated with 4 NHCl and 0.1 M sodium borate for 10 min each. After blocking with 10% NGS in 0.3% Triton X-100 in PBS (PBS-T), brain slides were incubated at 4 °C over night with the following primary antibodies diluted in blocking solution: mouse anti-BrdU (MoBU-1) (Thermo Fisher Scientific, #B35128, 1:100), mouse anti-Sox2 (abcam, ab79351, 1:50), rabbit anti-Sox2 (abcam, ab97959, 1:100) and rabbit anti-Tbr1 (abcam, ab31940, 1:200). On the next day, brain slides were incubated with the following secondary antibodies diluted 1:500 in blocking buffer for 1 h at room temperature: goat anti-mouse Alexa Fluor 555 (Cell Signaling, CST4409S) and goat anti-rabbit Alexa Fluor 488 (Cell Signaling, CST4412S). Nuclei were counterstained with DAPI (4′,6-diamidino-2-phenylindole, Roth, 1:1000 from a 1 mg/ml stock solution). After washing brain slides three times with PBS, they were mounted with Mowiol (Merck, 475,904-100GM) and stored at 4 °C in darkness.

### Golgi-Cox staining

Golgi-Cox staining was performed using FD Rapid GolgiStain Kit (FD NeuroTechnologie Inc, PK401). The freshly prepared mouse brains were treated with solution A, B and C according to the manufacturer’s recommendations. After that, brains were oriented with the forebrain directed to the top and embedded in a 6-well-plate in 3% Agarose (UltraPure™ Agarose, Product No. 16500500). Subsequently, the hardened agarose block was glued on a vibratome plate (Leica VT1200S, Leica Biosystems Nussloch GmbH, Nussloch, Germany) with Vetbond™ tissue glue (3 M™ Vetbond™ Gewebekleber, Product No. 10430774). Settings for cutting sections of necessary thickness were 180 µm thickness, 0,4 mm/s speed and 1 mm amplitude. Slices were caught in 1xPBS, transferred to a 12-well-plate and afterwards treated with solution D and E according to the manufacturer’s recommendations. After washing and drying, brain slices were transferred on a slide, mounted with Mowiol (Merck, 475,904-100GM) and stored at 4 °C in darkness for at least 24 h.

### DiI staining

For DiI staining, the crystalline and lipophilic fluorescence stain DiI (1,1'-Dioctadecyl-3,3,3',3'-Tetramethylindocarbocyanine Perchlorate, Thermo Fisher Scientific, D3911) was used. Formalin fixed brains were cut with a vibratome in 200 µm frontal slices. Respectively, two DiI crystals were applied in the somatosensory cortex and pyramidal cell layer of the CA1 region on both brain hemispheres with a thin insect needle. Next, tagged slices were rinsed with 1xPBS for maximum 16 h and subsequently fixed on a slide with 100 µl Fluoromount (Fluoromount-G®, SouthernBiotech, 0100–01). After 5 h of drying in the dark, slides were ready for microscopy.

### Imaging and image quantification

Pictures of Figs. 1, 2 IHC, 3, 4 H&E, 5 H&E, 6 IHC and supplement 1–5 and were taken with a NanoZoomer 2.0-HT slide scanner and processed in NDP.view 2.6.12 (Hamamatsu Photonics K.K., Hamamatsu, Japan). Pictures of Fig. 2 IF-P were taken with a Nikon ECLIPSE Ti2 microscope and integrated Nikon DS-Qi2 camera and processed in NIS-Elements AR 5.01 (Nikon Corp., Tokyo, Japan). Pictures of Figs. 4 and 5 Golgi-Cox staining were taken with a Olympus BX43 and integrated Olympus SC50 camera and processed with Olympus cellSens Entry 1.15 (Olympus Europa SE & Co. KG, Hamburg, Germany). Pictures of Figs. 4 and 5 DiI staining were taken with Leica TCS SP5 and 63 × objective (HCX PL APO Lbd. Bl. Oil, NA: 1.4–0.60, WD (mm): 0.1) (Leica Microsystems CMS GmbH, Wetzlar, Germany) and processed with Leica LAS AF 2.7.3 (Leica Microsystems CMS GmbH, Mannheim, Germany) as well as analyzed with Imaris 7.6.1 (Bitplane Oxford Instruments plc, Abingdon, UK) and MATLAB R2018b (The MathWorks, Inc., Natick, USA). General image editing was done with Adobe Photoshop Elements 15.0 (Adobe Systems Inc., San José, USA). All countings of cell marker expression, (z-) lengths and area measurements were done with ImageJ 1.50 (Wayne Rasband, National Institute of Health, USA). All quantifications were performed blinded in at least three animals (for exact numbers refer to figure legends). Thickness of the cerebral cortex was measured ten times through the whole cortex on frontal Golgi-Cox stained sections of P15 brains. For Golgi-Cox staining analysis, the parameters apical dendrite length, dendritic branches and spine density were measured in ten different neurons per brain in layer V of the cortex and pyramidal cell layer of the hippocampus on P15. For DiI staining microscopy and quantification of P15 brains, 50–60 pictures were taken in z-stacking mode (interval of 0.3–0.4 µm). Starting point of semi-automatic spine measurement for 3D-reconstruction was 50–100 µm distal from neuronal soma after the first branching point. Spine analyses of dendrites were executed on 50 µm length. For beginning of image processing, baseline subtraction was done by calculating (max value/10)*2. Afterwards, filament tracing was performed while using manual editing and segmented region of interest. Next, dendrite points diameter were scaled (largest 6.05 µm, thinnest 0.363 µm). For dendrite points, automatic starting point threshold plus removing seed points around starting points were used with a modulated threshold of seed points. Dendrite threshold of local contrast was set automatically with shortest distance from distance map algorithm. Spine points diameter was also set automatically allowing branch spines (seed points thinnest diameter 0,242 µm, maximum length 6,05 µm). For classifying spine points, seed point threshold was set automatically. Next, the spine diameter settings were modulated to logarithmic third lower line while also using the shortest distance from distance map algorithm. Then, different spine morphology classifications (stubby, mushroom, long thin and filopodia) were analyzed by spine density in [spines/µm]. Spine classification was based on Imaris 7.6.1 with logical data from MATLAB R2018b as shown in Fig. 4. All microscopy parameters and evaluation settings of semi-automatic filament tracing were performed in close cooperation with the UKE Microscopy Imaging Facility (UMIF). For analyses of mitotic spindles, division angles were determined by calculating the angle between mitotic spindles and the ventricular surface, using image J (“division angle relative to the VZ”) [16, 35].

### Proteomics and phosphoproteomics

#### FF and FFPE Tissue lysis and preparation for proteome analysis

The excised FFPE brain regions were deparaffinized with n-heptane followed by ethanol washes and lysed in 100 mM triethyl ammonium bicarbonate (TEAB) and 1% w/w sodium deoxycholate (SDC) buffer, boiled at 95 °C for 60 min antigen retrieval. Samples were sonicated with a probe sonicator for 10 pulses to destroy DNA/RNA.

The excised fresh frozen brain regions were lysed in 100 mM TAB and 1% w/w SDC buffer, boiled at 95 °C for 5 min and sonicated with a probe sonicator for 10 pulses. For proteome analysis 20 µg for phosphopeptide enrichment (FF only) 200 µg of protein of each sample was taken and disulfide bonds reduced in the presence of 10 mM dithiotreitol (DTT) at 60 °C for 30 min. Cysteine residues were alkylated in presence of 20 mM iodoacetamide at 37 °C in the dark for 30 min and tryptic digestion (sequencing grade, Promega) was performed at a 100:1 protein to enzyme ration at 37 °C over night. Digestion was stopped and SDC precipitated by the addition of 1% v/v formic acid (FA). Samples were centrifuged at 16.000 g for 5 min and the supernatant was transferred into a new tube. Samples were dried in a vacuum centrifuge.

#### Phosphopeptide enrichment with TiO_2_

A suspension of 1.2 mg TiO_2_ (5 µm particle size, Titansphere) in 100 µL loading buffer (5% triflouroacetic acid (TFA), 80% acetonitrile (ACN)) and 1 M glycolic acid was prepared. To each sample 100 µL was added to resuspend peptides. Samples were incubated at room temperature for 1 h at slight agitation (900 prm) in a thermo shaker (Eppendorf). Sample was transferred into a self-prepared C8 stage-tip (C8 disc, 3 M). The loading buffer was forced through the tip by centrifugation (5.000 g for 5 min), washed twice with 50 µL loading buffer and once with 50 µL of 0.1% TFA in 20% ACN. The tips were placed into new collection tubes and enriched peptides were eluted first with 50 µL 1% NH_4_OH in 30% ACN and twice with 50 µL 1% NH4OH in 50% ACN. Elution fractions were combined and dried in a vacuum centrifuge.

#### LC–MS/MS in Data Dependent and Data Independent mode

Fractions were resuspended in 0.1% formic acid (FA) and transferred into a full recovery autosampler vial (Waters). Chromatographic separation was achieved on a Dionex Ultimate 3000 UPLC system (Thermo Fisher Scientific) with a two-buffer system (buffer A: 0.1% FA in water, buffer B: 0.1% FA in ACN). Attached to the UPLC was a Acclaim PepMap 100 C18 trap (100 µm × 2 cm, 100 Å pore size, 5 µm particle size) for desalting a purification followed by a Acclaim PepMap 100 C18 analytical column (75 µm × 50 cm, 100 Å pore size, 2 µm particle size). Peptides were separation using a 60 min gradient with increasing ACN concentration from 2%—30% ACN. The eluting peptides from the fresh frozen samples (proteome and phosphoproteome), were analyzed on a quadrupole orbitrap ion trap tribrid mass spectrometer (Fusion, Thermo Fisher Scientific) in data dependent acquisition (DDA) and data independent acquisition (DIA). FFPE samples were analyzed on a quadrupole orbitrap mass spectrometer (QExactive, Thermo Fisher Scientific) in DDA monde.

For phosphopeptide analysis and FFPE brain regions, samples were analyzed in DDA mode, for the proteome analysis of the fresh frozen samples, randomly chosen samples from each time point were used to build a reference spectral library in DDA mode for data extraction of samples acquired in DIA mode. For DDA on the QExactive, the 15 most intense ions per precursor scan (1 × 10^6^ ions, 70,000 Resolution, 100 ms fill time) were analyzed by MS/MS (HCD at 25 normalized collision energy, 2 × 10^5^ ions, 17,500 Resolution, 50 ms fill time) in a range of 400 – 1200 m/z. A dynamic precursor exclusion of 20 s was used.

For DDA in the Fusion, the 12 most intense ions per precursor scan (2 × 10^5^ ions, 120,000 Resolution, 120 ms fill time) were analyzed by MS/MS (HCD at 30 normalized collision energy, 1 × 10^5^ ions, 15,000 Resolution, 60 ms fill time) in a range of 400 – 1300 m/z. A dynamic precursor exclusion of 20 s was used. For DIA, each sample was analyzed using a 32 sequential 25 Da fixed window method covering the mass range from 400 – 1,200 m/z. Per cycle, 2 precursor scans (2 × 10^5^ ions, 60,000 Resolution, 50 ms fill time, m/z range 390 – 1,210 m/z) and 32 MS/MS scans (HCD at 28 normalized collision energy, 1 × 10^5^ ions, 30,000 Resolution, 50 ms fill time) were performed. After the first precursor scan, 16 MS/MS scans were performed covering the precursor mass range from 400 to 800 m/z followed by the second precursor scan and another 16 MS/MS scans ranging from 800 to 1,200 m/z.

## Data analysis and processing

Acquired DDA LC–MS/MS data to generate a reference peptide spectra library for DIA data extraction were searched against the reviewed mouse SwissProt protein data base downloaded from Uniprot (release January 2019, 17,013 protein entries) using the Sequest algorithm integrated in the Proteome Discoverer software version 2.4. Mass tolerances for precursors was set to 10 ppm and 0.02 Da for fragments. Carbamidomethylation was set as a fixed modification for cysteine residues and the oxidation of methionine, pyro-glutamate formation at glutamine residues at the peptide N-terminus as well as acetylation of the protein N-terminus, methionine loss at the protein N-terminus and the Acetylation after methionine loss at the protein N-terminus were allowed as variable modifications. Only peptide with a high confidence (false discovery rate < 1% using a decoy data base approach) were accepted as identified. For FFPE proteome analysis, were also processed with Proteome Discoverer 2.4 with similar settings as above but using the reviewed mouse protein data base from Uniprot (release October 2020, 17,053 protein entries) in label free quantification mode with match between runs enabled, performing chromatographic re-calibration for precursors with a 5 min retention time tolerance, no scaling, and no normalization for extracted peptide areas. Peptide areas were summed to protein areas and used for quantitative analysis.

Proteome Discoverer search results from brain regions from fresh frozen samples were imported into Skyline software version 4.2 allowing only high confidence peptides with more than 4 fragment ions. A maximum of 5 fragment ions per peptide were used for information extraction from DIA files for peptides with a dot product of > 0.85. Peptide peak areas were summed to generate protein areas which were then used for relative abundance comparison. Protein areas were imported into Perseus software version 1.5.8 for statistical analysis [63].

The DDA data acquired for the phosphoproteome analysis searched against the reviewed human protein database downloaded from Uniprot (release January 2019 with 17,013 entries) processed with the Andromeda Algorithm included in the MaxQuant Software (Max Plank Institute for Biochemistry, Version 1.6.2.10). All samples were handled as individual experiments. The label-free quantification option with match between runs was used. Trypsin was selected as enzyme used to generate peptides, allowing a maximum of two missed cleavages). A minimal peptide length of 6 amino acids and maximal peptide mass of 6000 Da was defined. Oxidation of methionine, phosphorylation of serine, threonine and tyrosine, acetylation of protein N-termini and the conversion of glutamine to pyro-glutamic acid was set as variable modification. The carbamidomethylation of cysteines was selected as fixed modification. The error tolerance for the first precursor search was 20 ppm, for the following main search 4.5 ppm. Fragment spectra were matched with 20 ppm error tolerance. False discovery rate for peptide spectrum matches and proteins was set to 1%. For Quantification all identified razor and unique peptides were considered.

### Network and GO Term analyses

#### Processing and visualization of protein expression data

For both proteome and phosphoproteome, the respective ModificationSpecific.txt result file from MaxQuant was loaded into Perseus software (Max Plank Institute for Biochemistry, Version 1.6.2.3, Planegg, Munich) [63]. Samples were categorically annotated by genotype. The quantitative values for all proteins of proteome and peptides of phosphoproteome (peptides were filtered to display phosphopeptides only with ≥ 2 valid values in each group type) were transformed to log2 values and filtered by valid values. Columns of the newly generated matrix were normalized by subtracting the median. Next, two-sample Student’s t-test was performed with p-value truncation and p-value threshold of 0.05. Generated data was visualized via Z-score of rows using the median. A hierarchical clustering analyses was performed using the parameters “average linkage” and “Euclidean”.

#### Generation of network connection

Uniprot IDs of significantly altered proteins and peptide phosphorylation levels were copied into STRING 11.0 online database (Damian Szklarczyk, Swiss Institute of Bioinformatics and String Consortium 2020, Zurich, Swiss) [60] multiple protein search for mus musculus. Basic settings for meaning of network edges were evidence, active interaction sources were set to Textmining, Experiments, Database and Co-expression. The minimum required interaction score was 0.400. Afterwards, the generated string network was imported into Cytoscape via export section.

#### Cytoscape network and GlueGO Gene Ontology analysis

The generated network was imported into Cytoscape 3.8.2 (Trey Ideker, NRNB, NIH Biomedical Technology Research Center (BTRC), USA) [53] and its integrated application ClueGo 2.5.7 and CluePedia 1.5.7 (Bernhard Mlecnik, INSERM U872 Laboratory of Integrative Cancer Immunology, Paris, France) [8, 9]. For GlueGo analyses, functional analysis mode was selected. Marker list of mus musculus (10,090) was used and uniport IDs were loaded by name to fill marker list. The visual style was set to clusters to compare different cluster lists, such as increased abundance and decreased abundance cluster lists, and to underline specific terms and functions for each cluster. The ‘show genes shared between pathways/terms’ option was activated. For GO Term Biological Processes analyses, ClueGo settings of ontologies/pathways was GO BiologicalProcess-EBI-UniProt-GOA-ACAP-ARAP with #18,577 terms/pathways per ontology and 20,793 available unique genes from 08.05.2020 with all evidence. Only pathways with pV ≤ 0.05 were shown. Network specificity was medium (GO Tree Interval minimum level of 3 and maximum level of 8). GO Term/Pathway Selection was defined as minimum 3 genes and 4% of. GO Term/Pathway Network Connectivity (Kappa Score) was left preset by 0.4 (medium). For advanced statistical options, enrichment/depletion with two-sided hypergeometric test and Bonferroni step down pV correction was used also as standard setting. Grouping options were left in preset using GO term grouping with leading group term based on highest significance and Kappa score for 1 initial group size, 50% genes for group merge and 50% terms for group merge. The analog approach was used for GO Term KEGG and Reactome Pathways analyses (KEGG #324 (8760) from 08.05.2020, REACTOME Pathways #1666 (8584) from 08.05.2020) and GO Term Molecular Function and Cellular Component (MolecularFunction-EBI-UniProt-GOA-ACAP-ARAP #5333 (20,360) from 08.05.2020, CellularComponent-EBI-UniProt-GOA-ACAP-ARAP #1993 (20,819) from 08.05.2020) with all evidence. Biological Processes, Molecular Function and Cellular Component were used for network generation.

### Phosphosite analysis

For phosphosite analysis, the MS-based phosphoproteome dataset was filtered by significant genes using t-test p ≤ 0.05 and abs(x) > 0.5 referring to the expression data and network analysis approach. Then, a phosphosite search based on amino acid sequence of the relevant peptides was performed using the PhospoSitePlus® 6.5.8 database (Cell Signaling Technology, Inc., Danvers, USA) [24]. Next, upstream regulatory proteins of phosphosite hits from amino acid sequence were identified from the PhospoSitePlus® 6.5.8 database. The top three upstream regulatory protein hits were analyzed with regard to overlapping peptides using InteractiVenn for generating a venn diagram [20].

### Western blots

Fresh frozen cortex tissues were used to prepare 10% (w/v) homogenates in RIPA buffer (50 mM Tris–HCl pH 8, 150 mM NaCl, 1% NP-40, 0.5% Na-Deoxycholate, 0.1% SDS) freshly supplemented with 10 × protease inhibitor and PhosStop (Roche) on ice. Samples were homogenized using a Dounce homogenizer and then incubated on ice for 10 min. After a short vortex, homogenates were incubated for another 10 min prior to centrifugation at 12,000 g at 4 °C for 10 min. Total protein content was assessed by Bradford assay (BioRad). Supernatants of the brain homogenates were either further processed for SDS-PAGE or stored at − 80 °C for later usage.

For SDS-PAGE, samples were first denatured using 4 × Laemmli buffer (incl. 5% β-ME) and boiled for 5 min at 95 °C. Samples were then accordingly loaded on either precast Nu-PAGE 4–12% Bis–Tris protein gels (Thermo Fisher Scientific) or Any kD™ Mini-PROTEAN® TGX™ Precast Protein Gels (BioRad). After electrophoretic separation, proteins were transferred to nitrocellulose membranes (BioRad) by wet blotting. Membranes were then stained with Revert Total Protein Stain (LI-COR) following the manufacturer´s protocol to detect total amounts of protein. Membranes were subsequently blocked for 1 h with 1 × RotiBlock (Carl Roth) diluted in TBS-T and incubated overnight with the respective primary antibody (Suppl. Table 1) diluted in 5% BSA in TBS-T at 4 °C on a shaking platform.

Blots were subsequently washed with TBS-T for five times and then incubated for 1 h at RT with secondary antibody conjugates IRDye® 680RD and 800CW (Li-Cor) and then washed 5 × with TBS-T. The fluorescence signals were detected using an Odyssey CLX system (Li-Cor) according to the manufacturer instructions. Densitometric quantification was done using the Image studio lite software version 5.2.

Antibodies used for immunoblots as follows: rabbit Lin28A (Abcam, #63,740, 1:1000), rabbit β-III Tubulin (Cell signaling, #5568, 1:1000), rabbit GSK-3β (27C10) (Cell Signaling, #9315, 1:1000), mouse mAb Akt (pan) (40D4) (Cell Signaling, #2920, 1:2000), rabbit P-Akt (Ser473) (Cell Signaling, #9271, 1:1000), rabbit c-Myc (Cell Signaling, #5605, 1:1000), rabbit N-Myc (Cell Signaling, #51,705, 1:1000), rabbit Axin2 (Abcam, ab32197, 1:1000), rabbit Gli2 (Abcam, ab167389, 1:1000), rabbit APC (Abcam, ab15270, 1:1000), rabbit α-tubulin (Abcam, ab48389, 1:10,000), mouse HSC70 (Santa Cruz, sc-7298, 1:6000); blocking and buffer 5% BSA or milk in TBST. Secondary antibodies used as follows: donkey IRDye 680 RD Donkey anti-Rabbit IgG (Licor, #925–68,073, 1:10,000), donkey IRDye 800 CW Donkey anti-mouse IgG (Licor, #925–32,212, 1:10,000); blocking and buffer in 1 × Roti block.

### Modified open field test (OFT)

For analyzing anxiety, exploratory behavior and general activity of mice on P35, a modified open field test was performed within a dimmed h-temp polysulfon mouse cage (1284L EUROSTANDARD TYP II L, 365 × 207 × 140 mm, Hohenpeißenberg, Germany) as defined area. The mice were set in the top left corner and video measurements from 60 cm height started with the first movement of the mouse and ended after two minutes. Video material was analyzed with the organism video tracking software ToxTrac 2.84 (Umeå University supported by Kempestifteltelserna and IISD – Experimental Lakes Area Inc, Umeå, Sweden [51].

## Statistical Analyses

Statistics and data visualization were performed with GraphPad Prism 9.0 (GraphPad Software Inc., La Jolla, California, USA), except for heatmaps of non-hierarchical clustering analyses which were generated in Perseus software (Max Plank Institute for Biochemistry, Version 1.6.2.3) [63]. Protein and peptide expression values were compared as log2 data with two-tailed t-test (two groups). Means of two groups were compared using two-tailed, unpaired t-test. If the F-test defined significantly different variances, the Mann–Whitney test was performed instead of the t-test. Scatter dot plots are presented in mean ± SD, bars in mean ± SD, parts of whole in percentage and nonlinear regression (curve fit) in one phase decay. To display survival of mice, Kaplan–Meier plots were used and a log-rank (Mantel-Cox) test was performed to test for significance. For mitotic analysis, chi square test was used. All experiments concerning the phenotype of reported mice involved at least three mice (n = 3) per genotype (for exact numbers refer to figure legends). p values < 0.05 were considered to be significant. p values are shown in figures as ns = not significant (p > 0.05), * p < 0.05, ** p < 0.01, *** p < 0.001, **** p < 0.0001.

## Supplementary Information

**Additional file 1** **Figure S1**. Phenotype of *hGFAP-cre::lsl-Lin28A* mice (GL) compared to *hGFAP-cre *control mice (CTRL). (a) Scheme of mouse breeding and the *lsl-Lin28A* transgene. Mice carrying the *hGFAP-cre *promotor are crossed with *lsl-Lin28A* mice carrying the *CAGGS-loxP-PolyA-loxP-LIN28A(3x)-IRES-eGFP* sequence. Cre mediated recombination of loxP sites results in removal of the PolyA-Sequence (functional “STOP”-sequence). Consequently, LIN28A is constitutively overexpressed in neural progenitor cells of *hGFAP-cre::lsl-Lin28A *(GL) mice. (b-q) Fatemapping analyses of hGFAP-positive cells targeted by Cre using *hGFAP-cre::R26tdRFP*^*fl/+*^ mouse brains. Control forebrain (*lsl-R26tdRFP*^*fl/+*^) at P15 with high power images of the hippocampus region (c), stratum pyramidale (d) and isocortex (e). In contrast of the control, *hGFAP-cre::R26tdRFP*^*fl/+*^ mice displayed RFP-positive cells within the forebrain (j-m) at P15. High power images of the hippocampus (k) stratum pyramidale (l) and isocortex (m) are shown. Control cerebellum (*lsl-R26tdRFP*^*fl/+*^) at P15 with high power images of the granule cell layer (g), cerebellar layering (molecular cell layer, purkinje cell layer and granule cell layer) (h) and white matter (i). In contrast of the control, *hGFAP-cre::R26tdRFP*^*fl/+*^ mice displayed RFP-positive cells within the cerebellum (n-q) at P15. High power images of the granule cell layer (o) cerebellar layering (molecular cell layer, purkinje cell layer and granule cell layer) (p) and white matter (q) are shown. GL mice displayed no significant differences in body appearance (r) and body weight (s). GL mice displayed no significant difference in brain macroscopy (t), brain weight (u) and cortical thickness (v). Kaplan Meier analyses showing decreased survival of GL mice compared to CTRL mice (p=0.0028, log-rank test, n=83 for CTRL, n=58 for GL) (w). Scale bar in b is 1000 µm for b and j; scale bar in c is 500 µm for c and k; scale bar in d is 50 µm for d and l; scale bar in e is 200 µm for e and m; scale bar in f is 250 µm for f and n; scale bar in g is 50 µm for g and o; scale bar in h is 100 µm for h and p; scale bar in l is 50 µm for l and q. CTRL n=15, GL n=12 for s, CTRL n=83, GL n=58 for t. FB = forebrain, CB = cerebellum. P values are shown in figures as ns= not significant (p>0.05), * p<0.05, ** p<0.01, *** p<0.001, **** p<0.0001.
**Figure S2.** Organ macroscopy and microscopy of GL mice. (a) Organ macroscopy reveals no obvious macroscopic differences of heart, kidney, spleen, intestine, liver, pancreas and lung morphology (P100). (b-aq) No tumor formation or obvious change in histomorphology in respective organs of GL mice (w-ac) compared to CTRL (P30) (b-h). LIN28A staining of CTRL (i-v) and GL (ad-aq) showed no positive cells both in heart and kidney in CTRL (i-l) and GL (ad-ag). Positive cells in spleen and intestine of CTRL (m-p) and GL (ah-ak) mice. In liver, pancreas and lung, single LIN28A-positive cells were detected in GL mice only (al-aq) when compared to CTRL mice (q-v), indicating minor Cre-activity. Scale bar in b is 2,5 mm for i, w and ad; scale bar in c is 250 µm for d-h, k, m, o, q, s, u (CTRL), and x-ac, af, ah, aj, al, an, ap (GL); scale bar in insert j is 100 µm for j, l, n, p, r, t, v (CTRL) and ae, ag, ai, ak, am, ao, aq (GL); arrows mark LIN28A positive cells. (ar) Differential blood counts of 16-week-old CTRL and GL mice. GL mice showed significant higher hemoglobin (p=0.0286, unpaired t-test, n=4 for CTRL, n=4 for GL) and hematocrit (p=0.0197, unpaired t-test, n=4 for CTRL, n=4 for GL) values, significant lower absolute (p=0.0286, unpaired t-test, n=4 for CTRL, n=4 for GL) and relative (p=0.0286, unpaired t-test, n=4 for CTRL, n=4 for GL) neutrophil granulocyte values and significant higher monocyte (p=0.0129, unpaired t-test, n=4 for CTRL, n=4 for GL) and lymphocyte (p=0.0459, unpaired t-test, n=4 for CTRL, n=4 for GL) values. Pap-staining on P15 displays no significant changes in cytomorphology of GL blood cells when compared to CTRL (as, at). CTRL n=4, GL n=4 for ar. Scale bar in inset of as is 5 µm for blood cell insets in as and at
**Figure S3.** Analysis of mitotic phases and mitotic spindle orientation. (a) Representative pictures of mitotic phases. (b) Distribution of pro-/prometa-/metaphase, anaphase and telophase at the ventricular lining on E14.5 compared between CTRL and GL (p=0.6489, chi square test, each n=4). (c) Representative pictures of mitotic spindle orientation. (d) Scheme of mitotic spindle orientation by “division angle relative to the VZ”. (e) Distribution of mitotic spindle orientation in anaphase and telophase by vertical (60-90°), oblique (30-60°) and horizontal (0-30°) orientation at the ventricular lining on E14.5 compared between CTRL and GL (p=0.3325, chi square test, each n=4) (e). p/pm/m = prophase/prometaphase/metaphase, ana = anaphase, telo = telophase. Scale bar in a is 10 µm for a and c.
**Figure S4.** Cortical layering in GL mice. (a-aj) Immunostainings of CTRL and GL cortices at E14.5, P0 and P15. During development (E14.5) and at P0, SOX2 positive are found in the ventricular zone both in CTRL (a, m) and GL (d, p) mice. At P15 glia cells are also positive (y, ab). Both in CTRL (b, n) and GL (e, q) mice TBR2-positive cells were detected in the subventricular zone at E14.5 and P0, but not at later stages (z, ac). TBR1 marked early born neurons in in CTRL (c) and GL (f) mice at E14.5 and layer VI cortical neurons both in CTRL (o, aa) and GL (r, ad) mice at postnatal stages. CTIP2-positive layer V cortical neurons were detected both in CTRL (s, ae) and GL (v, ah) mice at P0 and P15. REEL (Reelin) positive Cajal-Retzius cells were found in the marginal zone layer both in CTRL (h, t, af) and GL (k, w, ai) mice. NeuN was not detected on E14.5 (i, l) but is seen in mature cortical neurons both in CTRL (u, ag) and GL (x, aj) mice at P0 and P15. Scale bar in a is 50 µm for a-l; scale bar in m is 100 µm for m-x; scale bar in y is 250 µm for y-aj; scale bar for panel a is 25 µm for all panels from a-aj.
**Figure S5.** Immunohistochemical analyses of hippocampal architecture in GL mice. (a-l) WFS and HUB stainings demonstrated similar formation of CA1 (WFS) and CA3 (HUB) areas in CTRL mice (a-f) and GL mice (g-l) at P15 despite granule cell dispersion in these regions. (m-aj) Immunohistochemical stainings of LIN28A, NeuN, REEL (Reelin), CTIP2, TBR2 and SOX2 of the stratum pyramidale in the CA1 region of CTRL (m-r) and GL (y-ad) and DG of CTRL (s-x) and GL (ae-aj). Strong LIN28A expression in the stratum pyramidale of CA1 region in GL (y, ae) mice in contrast to CTRL (m, s) mice. Similar to CTRL mice (n, t), GL mice (z, af) showed NeuN-positive neurons in the pyramidal cell layer and gyrus dentatus. Similarly, REEL-positive cells were detected in the molecular layer and Stratum oriens in CTRL (o, u) and GL mice (aa, ag). Strong CTIP2 expression in the DG of GL (ah) as well as in CTRL (v). Similar distribution of TBR2 positive cells in GL (ac, ai) mice compared to CTRL (q, w). Similar distribution of SOX2-positive cells in the subgranular zone of CTRL (r, x) and GL (ad, aj) mice. Similar distribution of Ki67-positive cells in the subgranular zone of CTRL (ak) and GL (al) mice on P15 and P30. Scale bar in a is 500 µm for a,d, g, j; scale bar in b is 200 µm for b, c, e, f, h, i, k, l; scale bar in m is 100 µm for m-r and y-ad; scale bar in s is 100 µm for s-x and ae-aj; scale bar in ak is 100 µm for ak and al. CA = cornu ammonis, DG = dentate gyrus; lines emphasize intactness of stratum pyramidale, arrows mark dispersion; PCL = pyramidal cell layer, GCL = granular cell layer, SGCL = subgranular cell layer
**Figure S6.** GL mice show hyperkinetic activity. Modified open field test of CTRL (a) and GL (b) mice on P35 analyzed with the animal tracking software ToxTrac displays significant higher values for the parameters distance travelled (c, p=0.0054, unpaired t-test, n=12 for CTRL, n=10 for GL), average speed (d, p=0.0042, unpaired t-test, n=12 for CTRL, n=10 for GL) and average acceleration (e, p=0.0099, unpaired t-test, n=12 for CTRL, n=10 for GL) in GL mice when compared to CTRL. Anxiety parameters such as exploration rate (f), time spent in center (g) and vertical activity (h) did not reveal significant differences. CTRL n=12, GL n=10 for c-h. P values are shown in figures as ns= not significant (p>0.05), * p<0.05, ** p<0.01, *** p<0.001, **** p<0.0001
**Figure S7.** Western Blot Analyses of selected Shh and Wnt targets. (a, b) Western blot and quantification of APC, GLI2, AXIN2, MYCN, MYC and LIN28A in cortices of GL and CTRL mice at P15. HSC70 and a-TUBULIN served as housekeeping proteins. Every sample number represents an individual mouse. Significantly increased LIN28A protein expression in GL mice compared to CTRL (p=0.0001, unpaired t-test, n=4 for CTRL, n=4 for GL). No significant changes in APC, GLI2, AXIN2, MYCN and MYC in GL mice compared to CTRL (p=0.2863, p=0.2859, p=0.1794, p=0.8252 and p=0.9872, respectively). P values are shown in figures as ns= not significant (*p*>0.05), * p<0.05, ** p<0.01, *** p<0.001, **** p<0.0001

**Additional file 2** Supplementary Table 1.

## Data Availability

LC–MS/MS data are available via ProteomeXchange with identifier PXD028044.

## Abbreviations

CTRL: Littermate control
ETMR: Embryonal tumor with multilayered rosettes
FF: Fresh frozen
FFPE: Formalin fixed and paraffine embedded
GL: *hGFAP-cre::lsl-Lin28A*
pHH3: Phosphorylated histone H3
RFP: Red fluorescent protein
VZ: Ventricular zone
