# Supplementary material for: Overexpression of Lin28A in neural progenitor cells in vivo does not lead to brain tumor formation but results in reduced spine density
Source: Acta Neuropathol Commun. 2021 Nov 20;9:185. doi: 10.1186/s40478-021-01289-1 (PMC8606090; doi:10.1186/s40478-021-01289-1)

Supplementary Figure 1

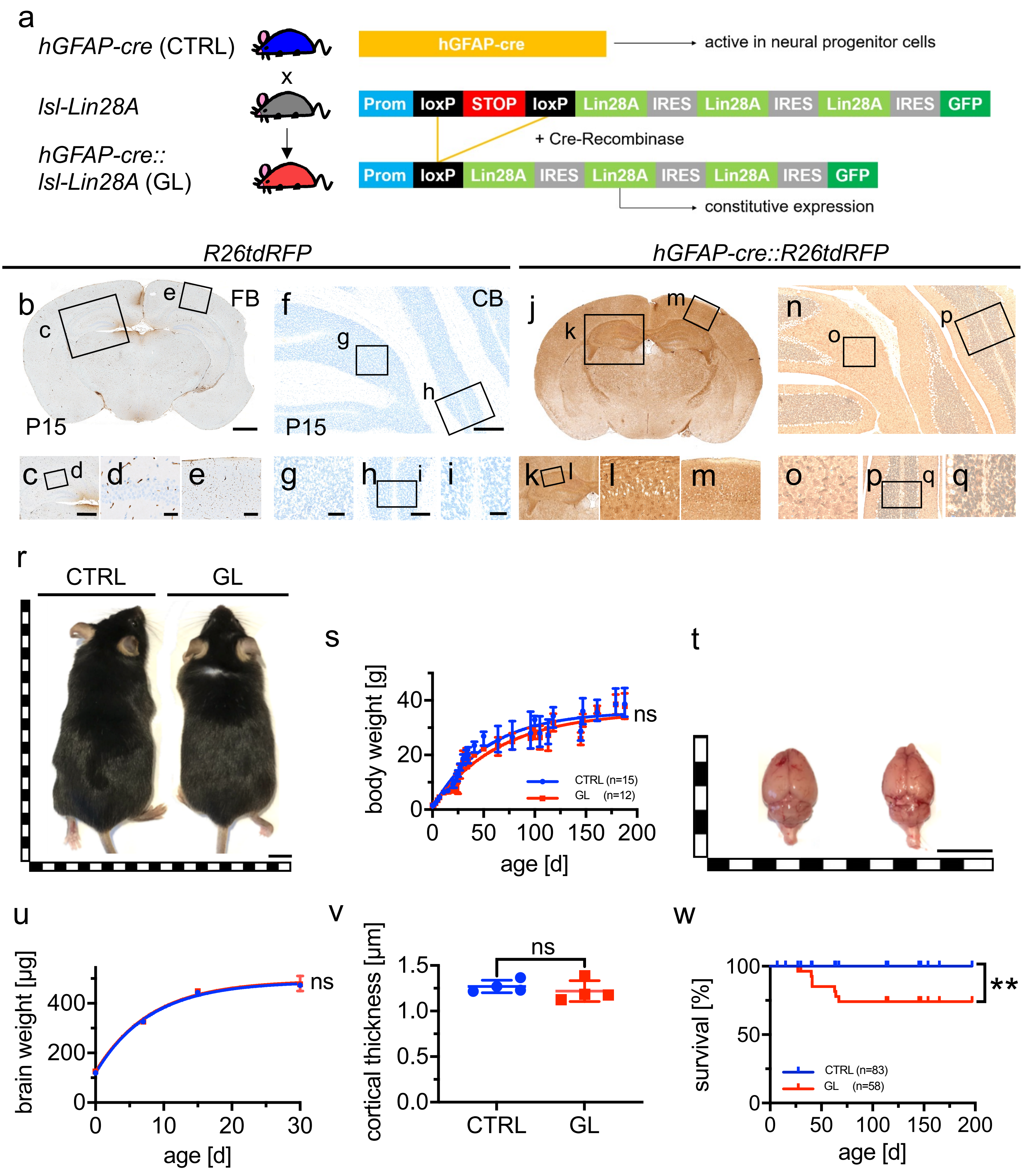

Supplementary Figure 2

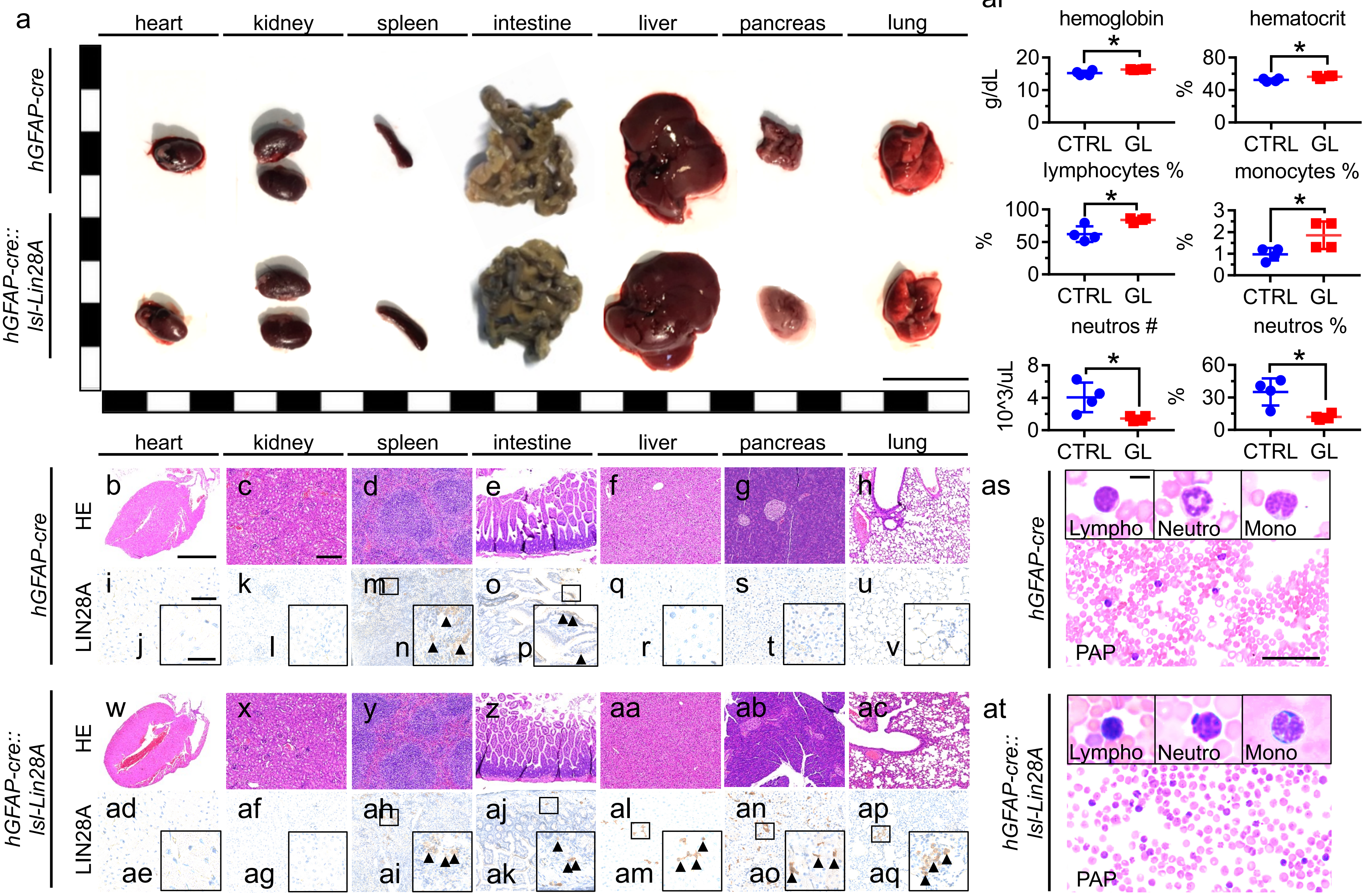

Supplementary Figure 3

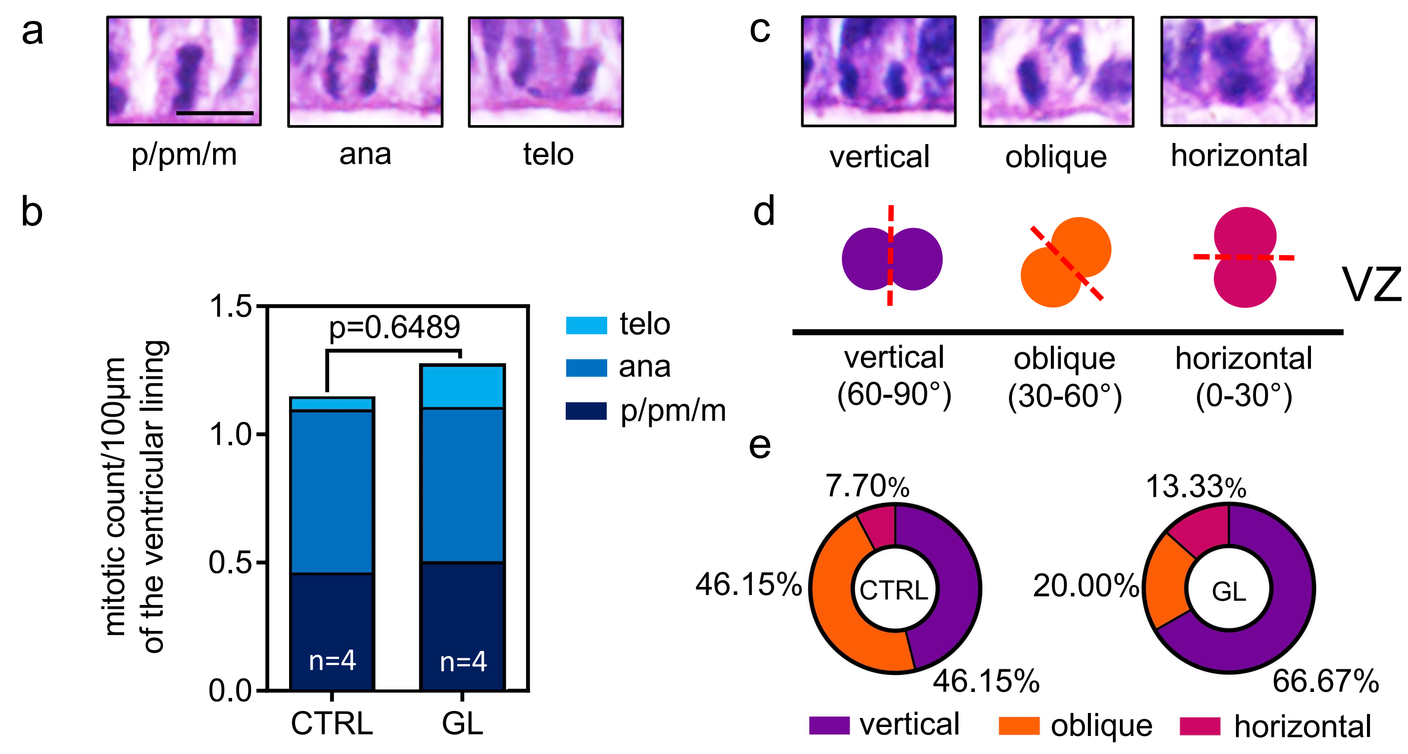

Supplementary Figure 4

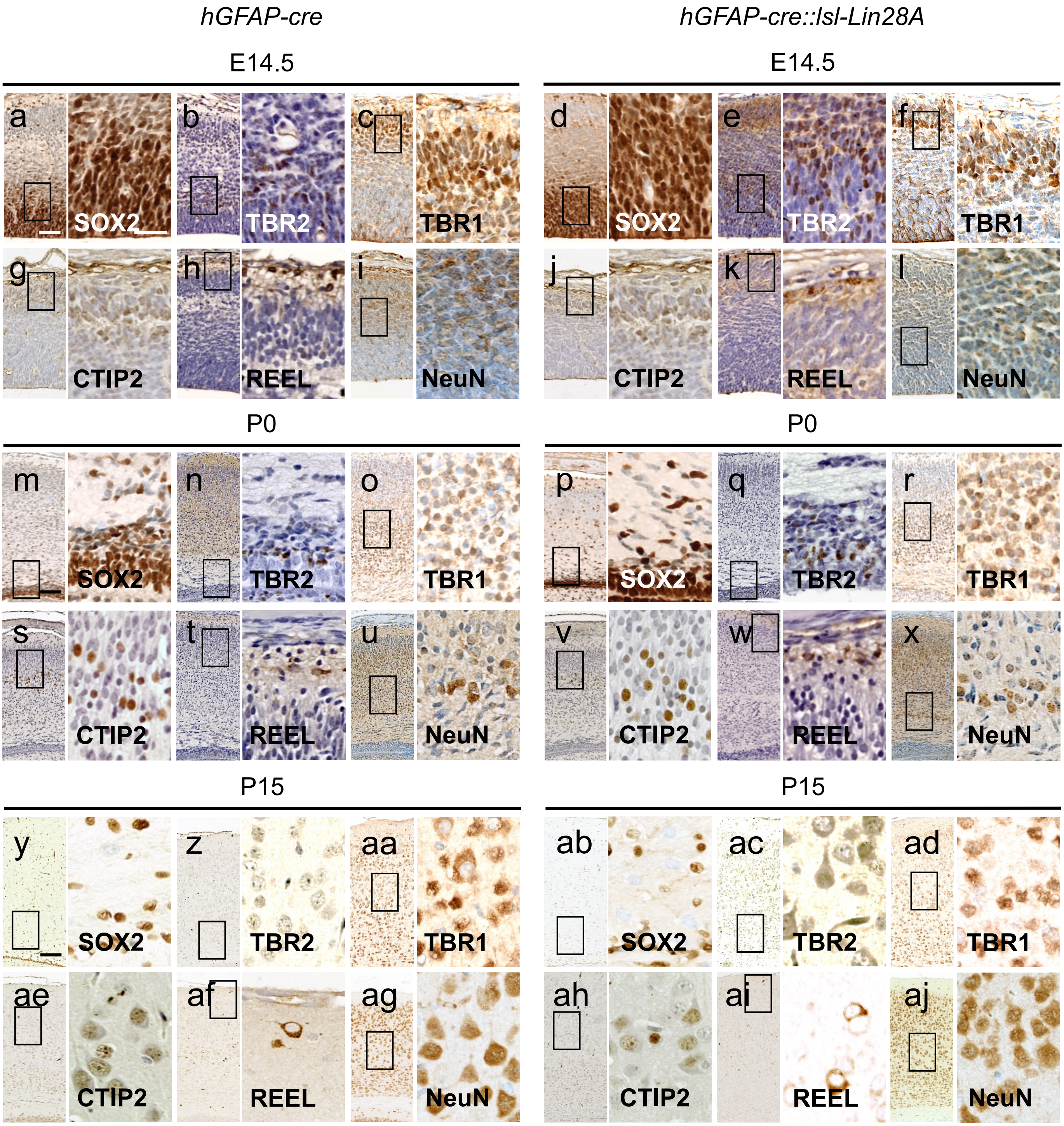

Supplementary Figure 5

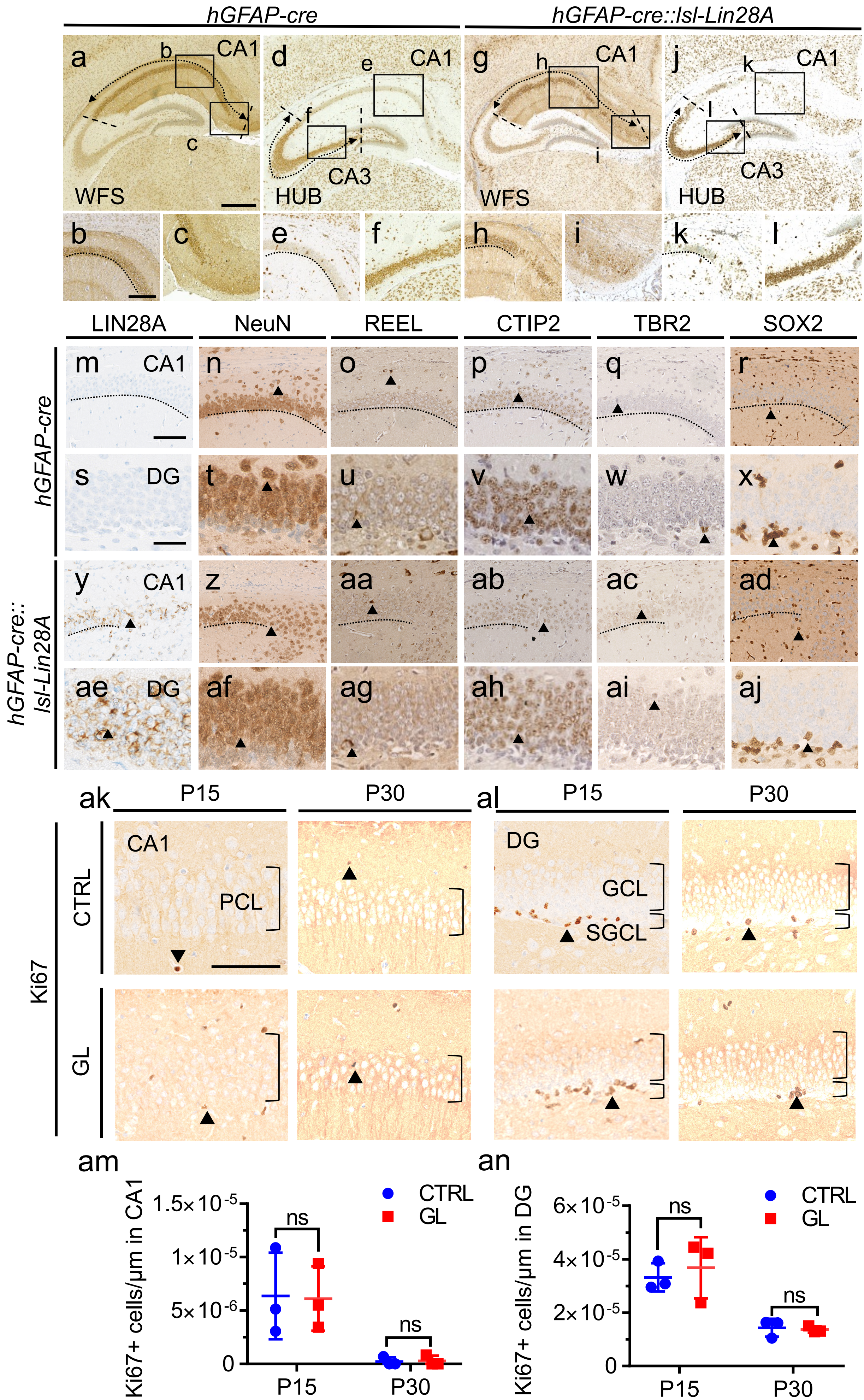

Supplementary Figure 6

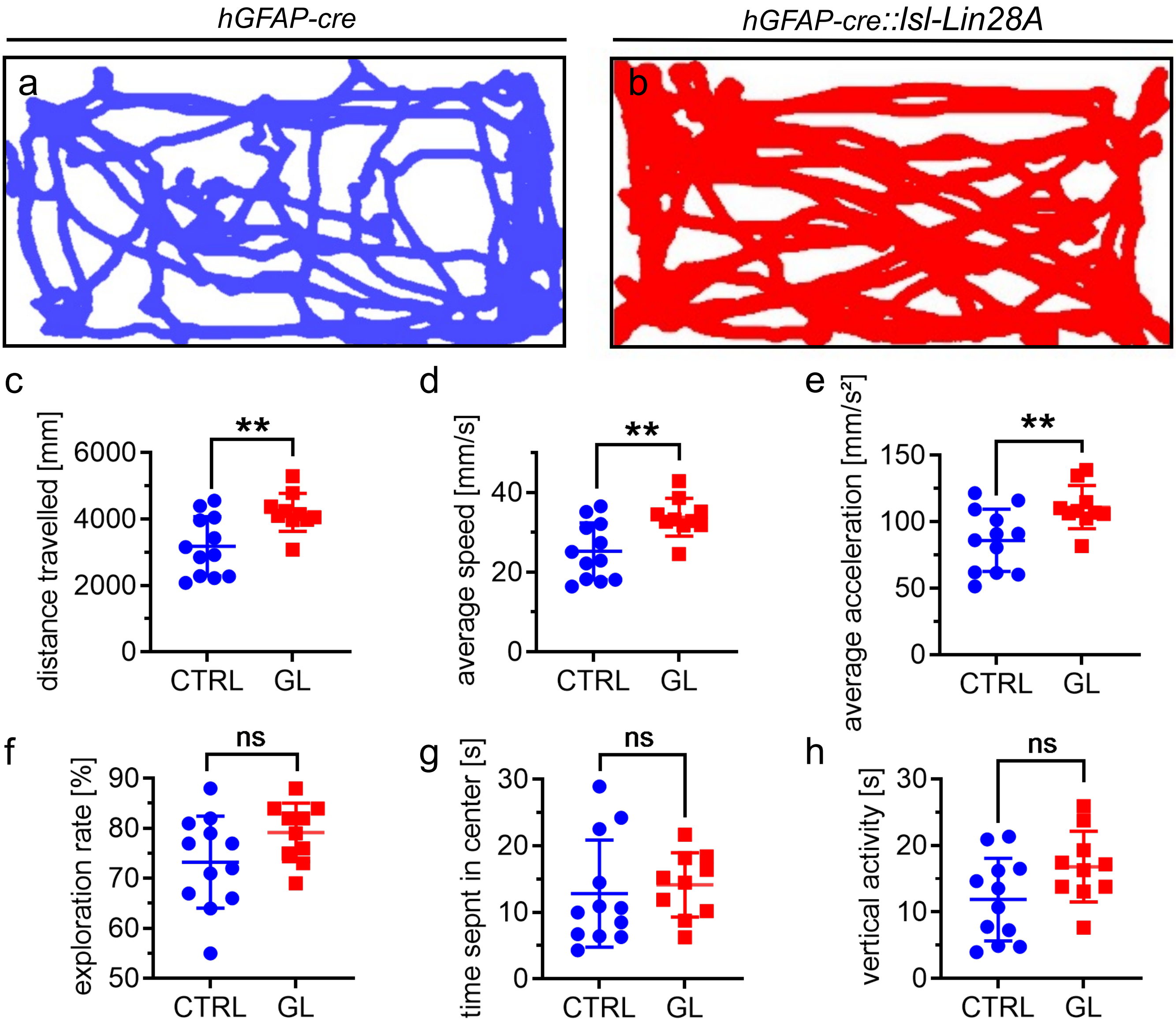

Supplementary Figure 7

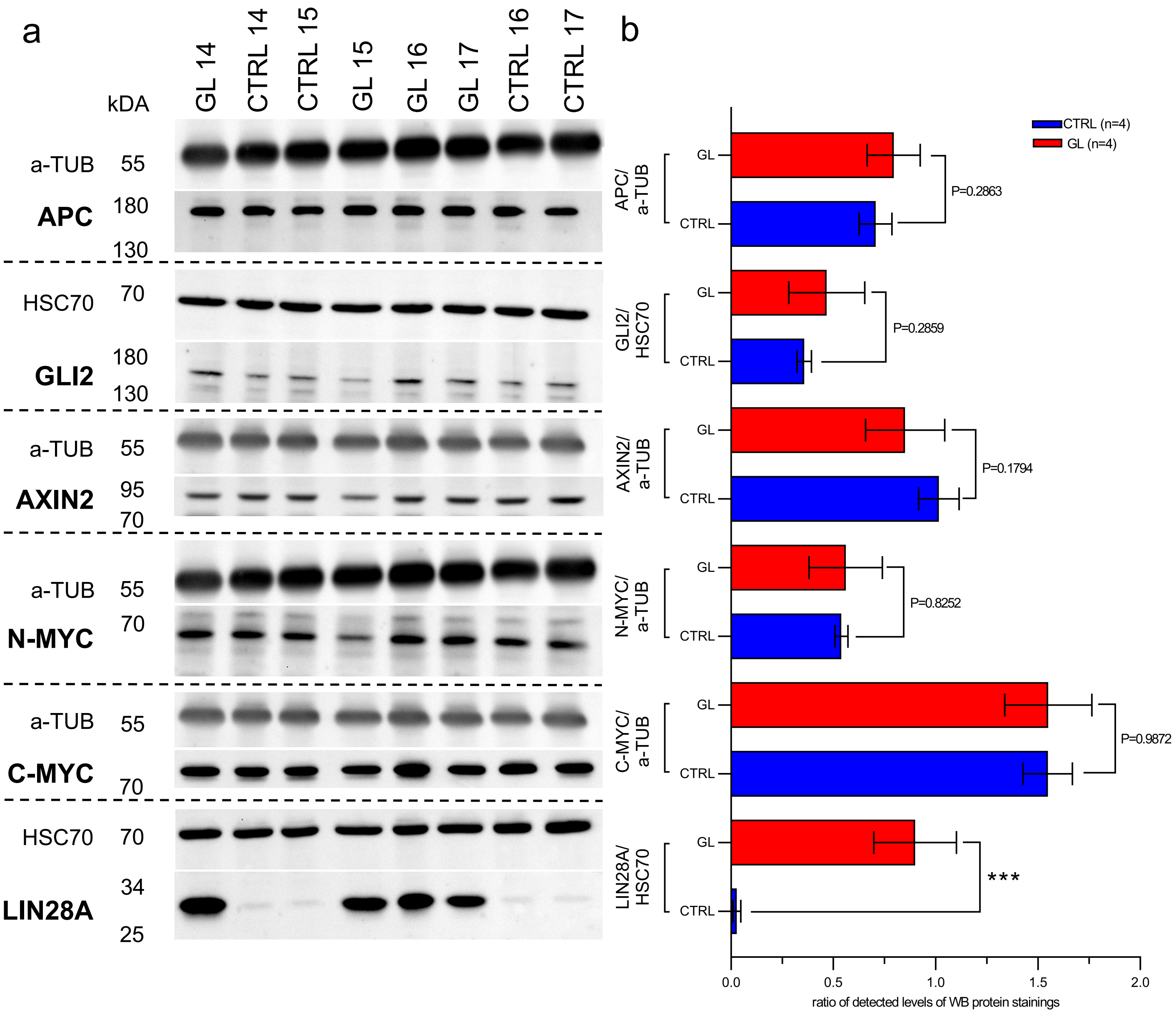

Supplement: Supplementary file 1 — Additional file 1 Figure S1. Phenotype of hGFAP-cre::lsl-Lin28A mice (GL) compared to hGFAP-cre control mice (CTRL). (a) Scheme of mouse breeding and the lsl-Lin28A transgene. Mice carrying the hGFAP-cre promotor are crossed with lsl-Lin28A mice carrying the CAGGS-loxP-PolyA-loxP-LIN28A(3x)-IRES-eGFP sequence. Cre mediated recombination of loxP sites results in removal of the PolyA-Sequence (functional “STOP”-sequence). Consequently, LIN28A is constitutively overexpressed in neural progenitor cells of hGFAP-cre::lsl-Lin28A (GL) mice. (b-q) Fatemapping analyses of hGFAP-positive cells targeted by Cre using hGFAP-cre::R26tdRFPfl/+ mouse brains. Control forebrain (lsl-R26tdRFPfl/+) at P15 with high power images of the hippocampus region (c), stratum pyramidale (d) and isocortex (e). In contrast of the control, hGFAP-cre::R26tdRFPfl/+ mice displayed RFP-positive cells within the forebrain (j-m) at P15. High power images of the hippocampus (k) stratum pyramidale (l) and isocortex (m) are shown. Control cerebellum (lsl-R26tdRFPfl/+) at P15 with high power images of the granule cell layer (g), cerebellar layering (molecular cell layer, purkinje cell layer and granule cell layer) (h) and white matter (i). In contrast of the control, hGFAP-cre::R26tdRFPfl/+ mice displayed RFP-positive cells within the cerebellum (n-q) at P15. High power images of the granule cell layer (o) cerebellar layering (molecular cell layer, purkinje cell layer and granule cell layer) (p) and white matter (q) are shown. GL mice displayed no significant differences in body appearance (r) and body weight (s). GL mice displayed no significant difference in brain macroscopy (t), brain weight (u) and cortical thickness (v). Kaplan Meier analyses showing decreased survival of GL mice compared to CTRL mice (p=0.0028, log-rank test, n=83 for CTRL, n=58 for GL) (w). Scale bar in b is 1000 µm for b and j; scale bar in c is 500 µm for c and k; scale bar in d is 50 µm for d and l; scale bar in e is 200 µm for e a [file 40478_2021_1289_MOESM1_ESM.pdf]
